# Contribution of nucleosome binding preferences and co-occurring DNA sequences to transcription factor binding

**DOI:** 10.1186/1471-2164-14-428

**Published:** 2013-06-28

**Authors:** Ximiao He, Raghunath Chatterjee, Sam John, Hector Bravo, B K Sathyanarayana, Simon C Biddie, Peter C FitzGerald, John A Stamatoyannopoulos, Gordon L Hager, Charles Vinson

**Affiliations:** 1 Laboratory of Metabolism, National Cancer Institute, National Institutes of Health, Room 3128, Building 37, 37 Convent Drive, Bethesda, MD 20892, USA; 2 Laboratory of Receptor Biology and Gene Expression, National Cancer Institute, National Institutes of Health, B602, Building 41, 41 Library Drive, Bethesda, MD 20892, USA; 3 Laboratory of Molecular Biology, National Cancer Institute, National Institutes of Health, Bethesda, MD 20892, USA; 4 Genome Analysis Unit, Genetics Branch, National Cancer Institute, National Institutes of Health, Bethesda, MD 20892, USA; 5 Center for Bioinformatics and Computational Biology, Biomolecular Sciences Bldg #296, University of Maryland, College Park, MD 20742, USA; 6 Department of Genome Sciences, University of Washington, Seattle, WA 98195, USA

**Keywords:** TFBS, Nucleosome, GR, c-Jun

## Abstract

**Background:**

Chromatin plays a critical role in regulating transcription factors (TFs) binding to their canonical transcription factor binding sites (TFBS). Recent studies in vertebrates show that many TFs preferentially bind to genomic regions that are well bound by nucleosomes *in vitro*. Co-occurring secondary motifs sometimes correlated with functional TFBS.

**Results:**

We used a logistic regression to evaluate how well the propensity for nucleosome binding and co-occurrence of a secondary motif identify which canonical motifs are bound *in vivo*. We used ChIP-seq data for three transcription factors binding to their canonical motifs: c-Jun binding the AP-1 motif (TGA^C^/_G_TCA), GR (glucocorticoid receptor) binding the GR motif (G-ACA---^T^/_C_GT-C), and Hoxa2 (homeobox a2) binding the Pbx (Pre-B-cell leukemia homeobox) motif (TGATTGAT). For all canonical TFBS in the mouse genome, we calculated intrinsic nucleosome occupancy scores (INOS) for its surrounding 150-bps DNA and examined the relationship with *in vivo* TF binding. In mouse mammary 3134 cells, c-Jun and GR proteins preferentially bound regions calculated to be well-bound by nucleosomes *in vitro* with the canonical AP-1 and GR motifs themselves contributing to the high INOS. Functional GR motifs are enriched for AP-1 motifs if they are within a nucleosome-sized 150-bps region. GR and Hoxa2 also bind motifs with low INOS, perhaps indicating a different mechanism of action.

**Conclusion:**

Our analysis quantified the contribution of INOS and co-occurring sequence to the identification of functional canonical motifs in the genome. This analysis revealed an inherent competition between some TFs and nucleosomes for binding canonical TFBS. GR and c-Jun cooperate if they are within 150-bps. Binding of Hoxa2 and a fraction of GR to motifs with low INOS values suggesting they are not in competition with nucleosomes and may function using different mechanisms.

## Background

Gene expression is ultimately controlled by the DNA sequence of the genome. The dramatically different DNA composition of proximal promoters in mammals [1] compared to yeast [2] and Drosophila [3] suggests that different mechanisms regulate gene expression in those organisms. Proximal promoters in yeast and Drosophila are AT rich and tend to be poorly-bound by nucleosomes both *in vitro* and *in vivo*[4-6] allowing easy access for transcription factors (TFs). In contrast, vertebrate promoters are often GC rich [7] and are well bound by nucleosomes *in vitro*[4,6,8]. *In vivo*, however, the GC rich promoters are instead bound by transcription factors and RNA polymerase II [4,5,9]. This observation lends support to a dynamic equilibrium switch mechanism where the promoter shifts from being bound by a nucleosome to being bound by TFs [3,10,11]. The kinetic interplay between these two states is mediated by the chromatin remodeling factors that disrupt, unwrap, and/or displace nucleosomes [10,12-14]. An extension of this competition model is a collaborative competition model where two TFs can bind to DNA independently but together can cooperate and displace a nucleosome if the two TFBSs are within 150-bps of each other [10,11,15-18]. The mechanistic details of this switch are complex with some TF being able to bind to DNA also bound by the histone octamer [19].

The determination of the *in vitro* binding of chicken nucleosomes to yeast genomic DNA allowed development of a scoring system that give an intrinsic nucleosome occupancy score (INOS) that indicates how well a nucleosome would bind any 150-bps of DNA [4,6]. This scoring system predicts that nucleosomes would bind CpG-rich regions well, which is consistent with what was observed [5,8] indicates credibility to the accuracy of the calculation. A general conclusion is that nucleosomes preferentially bind cytosine and guanine, sequences that often occur in clusters called CpG islands in mammalian genomes [5]. Hughes and colleagues have shown that in human samples, TF binding and DNase I hypersensitive sites (DHS) preferentially localize in genomic regions with high INOS [5].

In this study, we have focused on three TFs binding to their canonical TFBSs: c-Jun binding the AP-1 motif (TGA^C^/_G_TCA), glucocorticoid receptor (GR) binding the GR-like split 8-mer (G-ACA---TGT-C) [20-22], and Hoxa2 binding the homeobox Pbx motif (TGATTGAT) [23]. We show that nucleosomes are calculated to bind preferentially to both the GR and c-Jun motifs revealing an inherent competition between nucleosome and TF for binding. In contrast, the Hoxa2 motif is calculated to be less well bound by nucleosomes suggesting they are not in competition for binding the canonical motif [24]. Some Hoxa2 and GR, but not c-Jun, bound motifs have low INOS suggesting a second class of motifs that are not in competition with nucleosomes and may function using different mechanisms.

We used a logistic regression to evaluate the significance of these correlative observations and determined how well INOS and co-occurring DNA motifs could predict if a canonical motif would be bound by a TF. High INOS for canonical AP-1 motifs was a good predictor of c-Jun binding but co-occurring sequences was not predictive. For GR, in contrast, INOS was less predictive but co-occurring cis-motifs, (e.g., AP-1 or E-Box) was more predictive.

## Results

### GR and c-Jun proteins preferentially bind canonical DNA motifs in regions with high INOSs

**Figure 1:**
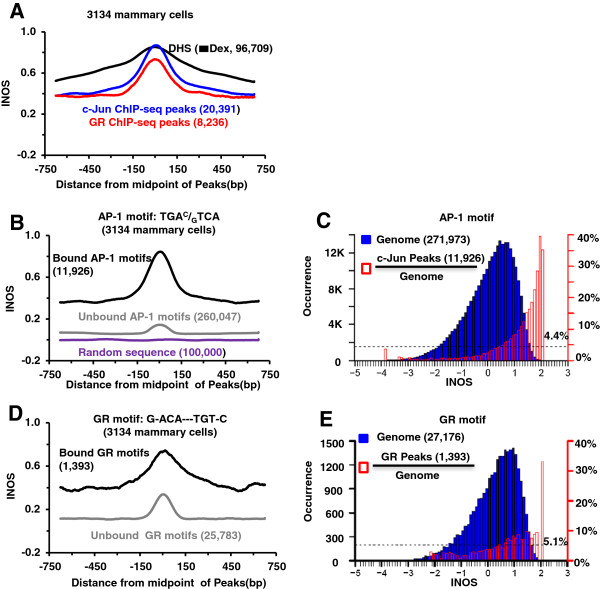
**Intrinsic nucleosome occupancy scores for regulatory sites.** (**A**) Average intrinsic nucleosome occupancy score (INOS) for 96,709 DNase I hypersensitive sites, 20,391 c-Jun peaks, and 8,236 GR peaks within ±750-bps from the center of the peak. (**B**) Average INOS near (±750-bps) canonical AP-1 motifs (TGA^C^/_G_TCA); 11,926 bound and 260,047 unbound AP-1 motifs are shown. 100,000 randomly selected sequences are shown as control. (**C**) The blue histogram shows the distribution of INOS for all 271,973 AP-1 motifs in the mouse genome and the red histogram shows the percent bound by c-Jun indicating preference for binding to the motifs with higher INOSs. 4.4% of all AP-1 sites are bound by c-Jun (black dashed line). (**D**) Average INOS near (±750-bps) canonical GR motifs (G-ACA---TGT-C); 1,393 bound and 25,783 unbound GR motifs are shown. (**E**) GR binds preferentially to GR motifs with higher INOSs. 5.1% of all GR sites are bound by GR (black dashed line).

Previous work has shown that dexamethasone induced GR protein binding preferentially occurs in DHS in the genome [20,21]. GR ChIP-seq data identified peaks for GR binding that were examined using MEME [25], DNA motif finding tools, and presented a position weight matrix for the enriched GR motif and the co-occurring AP-1 motif. We have extended this analysis and examined all DNA 8-mers in the form of the GR motif (N-NNN---NNN-N) termed a GR-like split 8-mer and calculated the enrichment of split-8-mers in GR peaks (Additional file 1: Figure S1A). Two sequences (G-ACA---TGT-C and G-ACA---CGT-C), which occur 27,176 and 7,394 times in the masked genome, are the most enriched (~20-fold) split 8-mers in the GR peaks. To exclude repetitive parts of the genome, we focused on the masked genome comprising ~55% of the genome [26]. Similar results are obtained when we examine the whole genome. The CG containing GR motif is not prominently seen in the published position weight matrix [21] reflecting it is rare in the genome. The variable enrichment of distinct sequences reflects the importance of studying individual sequence motifs instead of position weight matrices [27,28]. We also determined the enrichment of all 8-mers in the AP-1-like form (NNNNNNNN) in the 20,391 c-Jun peaks - the four most enriched 8-mers contained the canonical AP-1 7-mer (TGA^C^/_G_TCA) (Additional file 1: Figure S1B). INOS averages for GR peaks, c-Jun peaks, and DHSs spanning a 1,500-bp region have a maximum at the center of the peak with widths of ~300-bps (Figure 1A; Additional file 1: Figure S2) as shown previously for other mammalian transcription factors [5].

One way to evaluate how predictive high INOS is in TF localization in the genome would be to calculate an INOS for each nucleotide in the genome. However, to simplify the analysis, we calculated the INOS for all canonical motifs and compared this to ChIP-seq data that identify bound canonical motifs. Over half of the 20,391 c-Jun ChIP-seq peaks in mouse mammary 3134 cells contain a canonical 7-bp AP-1 motif. The average INOS across 1,500-bps for the c-Jun peaks with and without an AP-1 motif are similar (Additional file 1: Figure S3). The bound AP-1 motifs have significantly higher INOS values (~0.7) compared to unbound motifs (~0.1) (Figure 1B, Additional file 1: Figure S2) (p<2.2×10^-16^). Figure 1C presents a histogram summarizing INOS distribution for all AP-1 motifs in the genome and the percentage in c-Jun ChIP-seq peaks. c-Jun preferentially binds AP-1 motifs with high INOS, while only a few AP-1 motifs with low INOS are bound. 4.4% of canonical AP-1 motifs are bound by c-Jun, while up to ~30% of AP-1 motifs with high INOS (from 1.8 to 2.1) are bound by c-Jun (Figure 1B-C). To evaluate the significance of INOS for determining c-Jun binding to the AP-1 motif, we used a logistic regression. The percent of variance explained (PVE) and area under ROC curve (AUC) by INOS for c-Jun binding canonical motifs are 10.3% and 0.76 (Table 1, Additional file 1: Table S1A) indicating INOS is predictive of c-Jun binding. When we examine c-Jun binding to non-AP-1 sequences, INOS is less predictive (Additional file 1: Table S2A-B).

**Table 1:** **Modeling c**-**Jun**, **GR and Hoxa2 binding using a logistic regression**

**Evaluated parameters**	**AP**-**1 motif**			**GR motif**			**Pbx motif**		
	**(TGA**^**C**^**/**_**G**_**TCA)(11,926/271,973)**			**(G**-**ACA**---**TGT**-**C)(1,393/27,176**)			**(TGATTGAT)(638/59,802)**		
	**PVE(%)**	**CV err.**	**AUC**	**PVE(%)**	**CV err.**	**AUC**	**PVE(%)**	**CV err.**	**AUC**
**A1**. **Peak**	10.3	0.04	0.76	2.9	0.05	0.63	0.5	0.01	0.57
**A2**. **Background**	6.5	0.04	0.70	3.0	0.05	0.64	0.1	0.01	0.50
**A3**. **Relative peak**	1.9	0.04	0.62	0.2	0.05	0.53	0.6	0.01	0.57
**A**. **INOS** (**A1**+**A2**+**A3**)	**11**.**5**	0.04	0.77	**3**.**9**	0.05	0.66	**0**.**6**	0.01	0.57
**B**. **Overlap with CGIs**	0.9	0.04	0.53	0.1	0.05	0.51	0.0	0.01	0.51
**C**. **Cluster of canonical motifs**	5.5	0.04	0.66	0.6	0.05	0.51	0.1	0.01	0.51
**D**. **Co**-occurrence of 2nd motif*	**0**.**2**	0.04	0.51	**2**.**6**	0.05	0.57	**0**.**1**	0.01	0.51
**A**+**B**	12.3	0.04	0.78	3.9	0.05	0.66	0.6	0.01	0.57
**A**+**B**+**C**	13.9	0.04	0.79	4.2	0.05	0.67	0.7	0.01	0.58
**A**+**B**+**C**+**D**	14.0	0.04	0.79	6.7	0.05	0.70	0.8	0.01	0.58

Modeling the c-Jun binding to AP-1 motif (TGA^C^/_G_TCA), GR binding to GR motif (G-ACA---TGT-C), Hoxa2 binding to Pbx motif (TGATTGAT) were performed by the logistic regression using generalized linear models (GLM). *PVE* Percent of variance explained (%) = 1-(deviance/null.deviance). *CV err*. estimated 11-fold cross-validation prediction error, *AUC* Area under the ROC curve. *The Co-occurrence of 2nd motifs: the co-occurrence motif of AP-1 motif is GR motif (G-ACA---TGT-C), the co-occurrence of GR motif is AP-1 motif (TGA^C^/_G_TCA), the co-occurrence of Pbx motif is GR-like 8-mer (G-TGA---ATG-C). The PVEs of INOS and Co-occurrence of 2nd motif for AP-1, GR and Pbx motif are shown in bold to highlight the difference between the motifs.

The INOS for all canonical GR motifs (G-ACA---TGT-C) in the genome were also determined (Figure 1D, Additional file 1: Figure S2C). Like c-Jun, both peak and background INOS values are higher for the bound motifs than the unbound GR motifs (Figure 1D) (p<2.2×10^-16^) and have higher INOS near the peak (±150 bps) compared to nearby (±750 to ±150 bps). GR binds 5.1% of the canonical GR motifs, and preferentially binds to motifs in the genomic regions with higher INOSs (Figure 1E). Unlike c-Jun, some GR motifs with low INOS (−2 to −1) are bound (Figure 1C, Figure 1E, Additional file 1: Figure S2E) which may be indicative of a non-competitive model for TF and nucleosome binding [29]. Using the logistic regression, the PVE and AUC for INOS in the peaks to GR binding are 2.9% and 0.63, much less than 10.3% and 0.76 for c-Jun (Table 1, Additional file 1: Table S1A-B).

### **Two additional INOS parameters are predictive of GR and c**-**Jun binding to canonical motifs**

In addition to the INOS calculated when the canonical motif is at the center of the nucleosome-sized 150-bp genomic region (Peak value), we also examined the ‘Background’ value of INOS (±750 to ±150 bps) as well as the ‘Peak height’ or ‘Relative Peak’ value of INOS (the difference between the Peak and Background values) [5][30] (Figure 1A). Additional file 1: Table S3A characterizes AP-1 motifs based on ‘Background’ and ‘Relative Peak’ values revealing how these parameters can predict which motifs will be bound by c-Jun. For example, 14.4% of the 2,184 AP-1 motifs with “high” ‘Background’ and ‘Relative Peaks’ values are bound by c-Jun while zero of the 2,204 AP-1 sites with “low” ‘Background’ and ‘Relative Peaks’ values are bound. To extend the insights gained from averaging the INOS for all motifs, we examined individual values of ‘Relative Peaks’. 77% of bound AP-1 motifs have higher INOS near the motif (±150 bps) compared to adjacent DNA sequences (±750 to ±150 bps) while only 59% of unbound canonical AP-1 motifs have this trait (p<2.2×10^-16^). Similar but less dramatic results are observed for GR localization to canonical GR motifs (Additional file 1: Table S3B). 74% of canonical GR motifs have a higher INOS near the motif (±150 bps) compared to background (±750 to ±150 bps), while 66% of the unbound canonical GR motifs have this trait (p= 8.9 × 10^-10^).

A logistic regression indicates that the percent of variance explained (PVE) and AUC for ‘Background’ INOS for c-Jun localization to the canonical motif are 6.5% and 0.70, while the PVE and AUC of ‘Relative Peaks’ INOSs for c-Jun binding are only 1.9% and 0.62 (Table 1, Additional file 1: Table S1A). The least predictive factor was the relative peak value, which was previously reported as supporting a collaborative competition model [5,30]. For c-Jun binding to AP-1 canonical motifs, the combined PVE and AUC for these three parameters are 11.5% and 0.77 (Table 1, Additional file 1: Table S1A). For GR localization to canonical motifs, the PVE and AUC for Background INOSs are 3.0% and 0.64 but the PVE and AUC of Relative Peak INOSs are only 0.2% and 0.53 with the PVE and AUC of all three parameters are 3.9% and 0.66 (Table 1, Additional file 1: Table S1B).

### Nucleosomes preferentially bind GR and AP-1 motifs but not the Pbx motif

**Figure 2:**
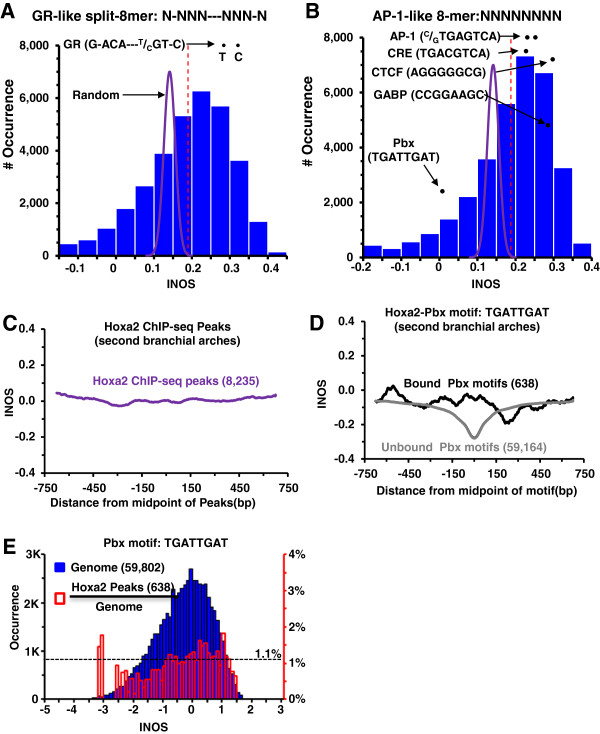
**The GR and AP**-**1 motifs are calculated to be well**-**bound by nucleosomes.** (**A**) Distribution of the INOS for 32,896 GR-like 8-mers (N-NNN---NNN-N). The INOS for each split 8-mer is calculated from 1,000 simulated 150-bp DNA sequences with the GR-like 8-mer in the center. The GR motifs are calculated to bind nucleosome better than the average GR-like 8-mer. The two GR motifs (G-ACA---^T^/_C_GT-C) are highlighted. Average INOS for all split 8-mers is shown as a dashed vertical red line. Distribution of INOS (0.141±0.015) for 32,896 random control set following Gaussian distribution is shown in purple. (**B**) Distribution of the INOS for all continuous 8-mers (NNNNNNNN). (**C**) Average INOS for 8,235 Hoxa2 ChIP-seq peaks [23]. (**D**) Average INOS near (±750-bps) canonical Pbx motifs (TGATTGAT); 638 bound as observed in the Hoxa2 ChIP-seq peaks and 59,164 unbound Pbx motifs are shown. (**E**) The blue histogram shows the distribution of INOS for all 59,802 Pbx motifs and the red histogram shows the percent bound by Hoxa2.

c-Jun and to an extent GR, prefer to bind motifs embedded in 150-bp long nucleosome-sized regions with high INOS, however the contribution of the TFBS itself to INOS of the entire 150-bps remains unclear. To address this issue, for each motif, we calculated the INOS for 1,000 simulated random 150-bp DNA sequences with 42% GC content as occurs in the mouse genome with the motif at the center. We examined all 32,896 GR-like 8-mers (N-NNN---NNN-N) and AP-1-like 8-mers (NNNNNNNN), as well as a control set where motifs at the center were also randomized (Figure 2A-B). The canonical GR motif (G-ACA---^T^/_C_GT-C) has a higher score than the average INOS or the control set (p= 1.5 × 10^-33^) suggesting that nucleosomes preferentially bind the GR motif. When we examined continuous 8-mers, a distribution was observed with many classic motifs, including AP-1, having higher INOS than random, similar to what was observed for GR (Figure 2B).

We next used the logistic regression to evaluate the contribution of the 150-bps without the TFBS to INOS. To examine this, we maintained the DNA sequences surrounding the motif and replaced the motif by random bases with GC content of 42%, and then calculated INOS. Excluding the TFBS from the calculation of INOS decreases the PVE of INOSs from 10.3% to 7.4% and AUC from 0.76 to 0.72 for c-Jun localization indicating that both the AP-1 motif and additional sequences drive preferential nucleosome binding (Additional file 1: Table S1A). Similar results (the PVE decreases from 2.9% to 2.7%, AUC decreases from 0.633 to 0.632) were observed for GR motifs (Additional file 1: Table S1B).

Some TFBSs, such as Homeobox Pbx (TGATTGAT) motif, have low INOS (p=4.1×10^-21^) and thus are not competing with nucleosomes for binding *in vivo* (Figure 2B-C). Examining published Hoxa2 ChIP-seq peaks from mouse secondary bronchial arches [23] (Figure 2C), we observed that motifs with both high and low INOS were bound. We examined all 59,802 occurrence of the Pbx motif in the genome and calculated INOSs for both the 638 bound and 59,164 unbound Pbx motifs (Figure 2D-E, Additional file 1: Figure S1C). The bound TFBS with low INOS suggest the homeobox protein is not in competition with nucleosomes for binding to DNA as previously observed for TFs that bind yeast promoters [29].

### GR binding and open chromatin

We correlated GR binding with the presence of a DHS to better understand the relationship between TF binding, the presence of a canonical motif, and nucleosome remodeling. GR peaks were classified into three groups, i.e. 71% in pre-programmed DHSs (DHSs observed in the absence of dexamethasone), 24% in re-programmed DHS (new DHSs induced after activation of GR by dexamethasone), and the remaining 5% in the regions that are not DHSs, which we term un-programmed DHS. GR motifs in all three groups have similar INOS with the pre-programmed DHS having slightly higher values (Additional file 1: Figure S2A and S2C) 9% of the GR peaks in pre-programmed DHSs contain a canonical GR motif, 34% of GR peaks at re-programmed DHSs contain a canonical GR motif, while 48% of GR peaks not in DHSs contain a canonical GR motif (Table 2, Additional file 1: Figure S2A and S2C), suggesting a canonical motif facilitates GR binding in chromatin (p<2.2×10^-16^). Inclusion of the experimentally determined DHSs to the logistic regression, increased the PVE for c-Jun and GR binding to 56.5% and 39.3% respectively, and increased AUC to 0.96 and 0.88 (Additional file 1: Table S1A-B).

**Table 2:** **Effect of A**-**FOS on GR binding to the GR motif and 1**-**bp variants**

**GR Motifs**	**#in Genome**	**#in Peaks(%of Peaks with the Motif)**	**%With AP**-**1(%in c**-**Jun Peaks)**	**+AP**-**1**	**---AP**-**1**
				**%A**-**Fos**	**%A-Fos**
		**Pre**-**Programmed** (**5**,**865 Peaks**)			
**G**-**ACA**---**TGT**-**C**	27,176	523 (9%)	23% (80%)	44%	15%
**G**-**ACA**---**CGT**-**C**	7,394	131 (2%)	23% (80%)	38%	20%
**1**-**bp Variants**	575,842	1,719 (30%)	31% (86%)	54%	28%
		**Re**-**Programmed** (**1**,**946 Peaks**)			
**G**-**ACA**---**TGT**-**C**	27,176	665 (34%)	14% (44%)	45%	9%
**G**-**ACA**---**CGT**-**C**	7,394	134 (7%)	10% (23%)	39%	4%
**1**-**bp Variants**	575,842	806 (41%)	18% (33%)	50%	11%
		**Un**-**Programmed** (**425 Peaks**)			
**G**-**ACA**---**TGT**-**C**	27,176	205 (48%)	6% (0%)	58%	8%
**G**-**ACA**---**CGT**-**C**	7,394	29 (7%)	7% (0%)	50%	4%
**1**-**bp Variants**	575,842	186 (44%)	9% (6%)	41%	11%

Effect of A-FOS on GR binding to the GR motif and 1-bp variants depends on the presence of an AP-1 motif within 150-bps. GR ChIP-seq peaks are placed into three groups, pre-programmed, re-programmed, and un-programmed. Column 1: GR motifs. Column 2: # of occurrences of each motif in the genome. Column 3: # of motifs bound by GR. In brackets is the % of peaks that contain the motif. Column 4: % of peaks containing a GR motif containing an AP-1 motif within 150-bps. In brackets is the % of GR motifs contain an AP-1 motif that are bound by c-Jun. Column 5: % of GR motifs containing an AP-1 motif nearby that loses GR binding after inhibition of c-Jun binding by the dominant negative A-FOS. Column 6: % of GR motifs without a nearby AP-1 motif that lose GR binding after inhibition of c-Jun binding by the dominant negative A-FOS.

**Figure 3:**
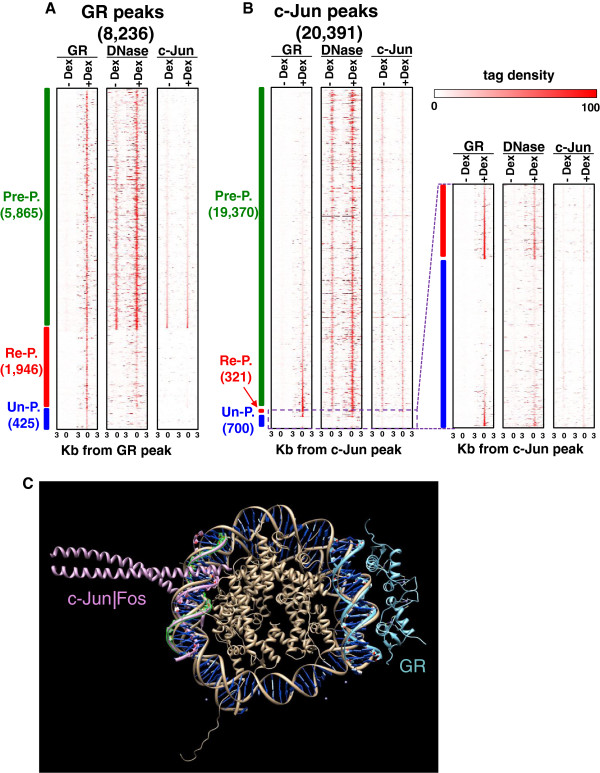
**Chromatin accessibility of GR and AP**-**1 motifs in 3134 cell line *in vivo.*** (**A**-**B**) Density of sequenced tags for GR, c-Jun ChIP-seq and DNase I-seq were counted in 3-Kb up and downstream of the center of (**A**) the GR ChIP-seq peaks and (**B**) the c-Jun ChIP-seq peaks. GR peaks are placed in three groups, pre-programmed DHS peaks, re-programmed DHS peaks, and GR peaks that are not in a DHS peak termed as un-programmed. The bin size is 300-bps and slide window is 150-bps. Sequence reads for GR, DHS, and c-Jun are normalized w.r.t the total tag-density. Re-programmed and un-programmed c-Jun peaks are enlarged to better present the tag-density pattern. (**C**) Overlapping crystal structures for the nucleosome (PDB ID: 1AOI), GR (1R4O) and c-Jun|Fos (1FOS) showing GR can bind the nucleosome-occluded DNA while c-Jun|Fos has steric hindrances.

To more clearly examine GR binding in the un-programmed peaks, we compared the sequencing tag-density of GR and c-Jun binding, and the DHS signal within 3-Kb of the peak (Figure 3A-B). In the 425 un-programmed GR peaks, a clear signal for GR binding was observed yet there was a less signal for the DHS. In contrast to GR, more than 95% c-Jun binding is at pre-programmed DHSs. Only 1.5% of c-Jun binding is in re-programmed DHSs (n = 321) and in all these cases, a clear signal of tag density is observed for c-Jun, GR and DHS (Figure 3B). 42% of c-Jun peaks in re-programmed DHSs have a canonical AP-1 motif and 93% are bound by GR suggesting these two proteins are acting together resulting in more open chromatin. 3% of c-Jun peaks are in un-programmed DHSs but little tag density is observed for either c-Jun or DHSs in contrast to the GR tag density at un-programmed peaks suggesting that GR has a higher propensity to bind in chromatin (Figure 3B, Additional file 1: Figure S2B, Figure S2D).

An alternative method to evaluate GR and c-Jun binding is to compare normalized tag density within 150-bps for GR and c-Jun peaks containing canonical motifs with DHS reads (Additional file 1: Figure S4A-B). We examined the AP-1 8-mer (ATGAGTCA) for a more accurate comparison with the GR motif 8-mer (G-ACA---TGT-C). The slope of c-Jun reads against DHS reads at the non-DHS regions is much higher than for GR implying GR can bind to the non-DHS regions better than c-Jun. Visualization of tag-density profiles within the 3-Kb upstream and downstream of the canonical GR motifs and AP-1 8-mer (ATGAGTCA) supports GR binding in non-DHS regions (Additional file 1: Figure S4C-D). X-ray crystal structures of GR (PDB ID: 1R4O) and AP-1 (PDB ID: 1FOS) proteins bound to their canonical DNA motifs were overlaid with an X-ray structure of the histone octamer (PDB ID: 1AOI) bound to DNA (a nucleosome) using the program Chimera [31] to create a physical model of these proteins bound to the same DNA. No physical clashes were observed when GR and the histone octomer bind the same DNA as has been experimental shown [19], while clashes are observed when both AP-1 and the histone octamer bind the same DNA (Figure 3C).

We next examined how the three classes of GR motifs at pre-, re-, and un-programmed DHSs correlate with expression of nearby genes (Additional file 1: Table S4A-C). GR peaks with canonical motifs at pre-programmed DHS tend to activate nearby genes (34%) compared to canonical motifs in un-programmed DHS (14%). In contrast, un-programmed peaks without a motif tend to be more activating (39%) in comparison to the pre-programmed peaks (21%).

### Clusters of identical TF motifs are better bound

To identify additional sequence properties besides INOS that are predicative of TF binding to canonical motifs, we examined co-localization with the same sequence (homotypic clusters [32]) or a second sequence. Canonical motifs were placed into bins depending on the distance to the nearest canonical motif. Additional file 1: Figure S5A shows, for example, there are only ~300 GR motifs with a second GR within 150-bps and 18% are bound by GR. This increase relative to the ~5% bound for a single GR motif may be because they are called as a single peak. In contrast, there are ~1,000 GR motifs that do not have nearest neighbor within 100,000-bps and only 3% are bound (Additional file 1: Figure S5A). Similarly, 18% of the ~20,000 AP-1 motifs that are within 150-bps of a second AP-1 motif are bound by c-Jun (Additional file 1: Figure S5B). Adding the clustering of GR motifs into the logistic regression increased the PVE from 3.9% to 4.2% and AUC from 0.66 to 0.67 (Table 1, Additional file 1: Table S1B).

### An AP-1 motif within 150-bps of a GR motif triples GR binding

**Figure 4:**
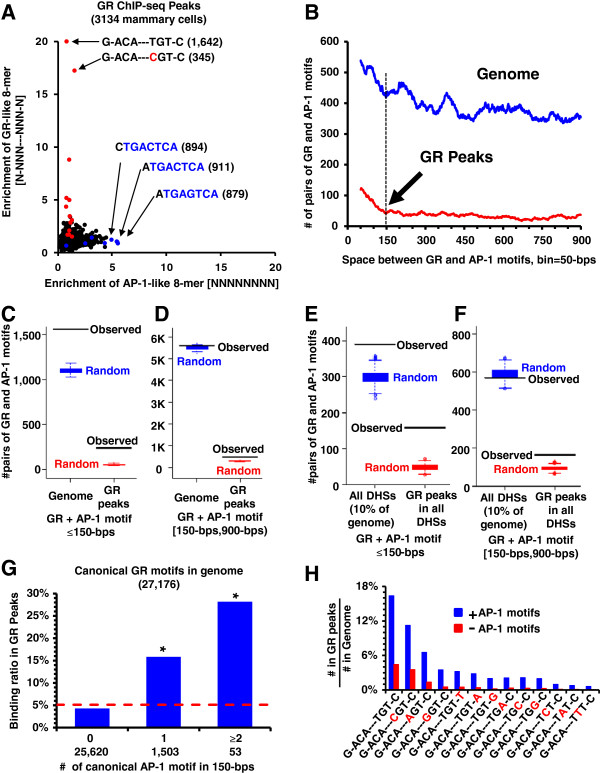
**GR and AP**-**1 motifs co**-**occurrence in the genome and GR peaks.** (**A**) Enrichment of split 8-mers (N-NNN---NNN-N) vs. all continuous 8-mers in the GR ChIP-seq peaks. Red dots: the canonical GR motif and 1-bp variants (mismatch in red), Blue dots: the 8-mers with AP-1 motif.(**B**) The co-occurrence of canonical GR and AP-1 motifs at distances up to 900-bps in masked mouse genome (blue) and GR peaks (red). The bin size is 50-bps with 1-bp sliding window. (**C**) The co-occurrence of canonical GR and AP-1 motifs within a nucleosome range (<150-bps) is statistically significant in both the genome (p=3×10^-53^) and GR peaks (p=4×10^-162^). (**D**) Same as (**C**) but for GR and AP-1 motifs separated by 150-bps to 900-bps. Their co-occurrences in the genome are close to expected occurrences (p=0.08) but enriched in GR peaks (p=4×10^-49^). (**E**) The co-occurrence of canonical GR and AP-1 motifs within a nucleosome range (<150-bps) is statistically significant in both the DHS regions (p=2×10^-7^) and GR peaks (p=1×10^-179^). (**F**) Same as (**E**) but for GR and AP-1 motifs separated by 150-bps to 900-bps. Their co-occurrences in the DHS regions are close to expected occurrences (p=0.77) but enriched in GR peaks (p=3×10^-12^). (**G**) Number of GR motifs with 0, 1, or 2 or more AP-1 motifs within 150-bps and y-axis represents percent in the GR peaks. The * denotes the statistical significance (p<1×10^-10^) of co-occurring GR and AP-1 motifs being bound by GR protein compared to GR motifs without an AP-1 motif. (**H**) Percent of canonical or 1-bp variants of GR motifs with (blue) or without (red) canonical AP-1 motifs within 150-bps in the GR peaks. Motifs are presented by their enrichment in the GR peaks.

Previously, the AP-1 motif was shown to enrich in GR peaks [21]. To identify a second sequence enriched in GR peaks, we compared the enrichment of all GR-like split 8-mers (N-NNN---NNN-N) to continuous 8-mers (NNNNNNNN) (Figure 4A). The three most enriched continuous 8-mers are AP-1 canonical motifs (>5-fold enriched).

The effect of the distance between AP-1 and GR motifs on GR binding was examined (Figure 4B-D). In the masked genome, there is a decrease in the number of GR and AP-1 motif pairs as the distance between them increases from 0-bps to 900-bps (Figure 4B). There are 1,566 occurrences of an AP-1 motif within 150-bps of a GR motif in the genome, much higher than expected (p=3×10^-53^) calculated using *in silico* random sampling in the genome (Figure 4C-D). When we examined GR ChIP-seq peaks, GR motifs with a nearby AP-1 motif are better bound, with a clear inflection at 150-bps. At shorter distance, there is no unique spacing between the two motifs implying no direct physical interaction between GR and c-Jun (Additional file 1: Figure S6A) with GR and AP-1 motifs that are closer together being better bound (Figure 4B). If the two motifs are between 150-bps to 900-bps, the observed occurrences in the genome and GR peaks is closer to expected occurrences. We also examined the co-occurrence of GR and AP-1 motifs in all DHS regions (~10% of mouse genome), and the similar results are observed (Figure 4E-F). For GR motifs with an AP-1 motif within 150-bps, GR preferentially binds motifs with higher INOS (Additional file 1: Figure S6B). The PVE and AUC by the co-occurrence of an AP-1 motif within 150-bps of a GR motif are 2.6% and 0.57, and the addition of INOS to AP-1 co-occurrence increased the PVE to 6.7% and the AUC to 0.70 (Table 1). There are 53 GR motifs with two or more AP-1 motifs within 150-bps in the genome and 28% of them are bound suggesting that the AP-1 motifs can act additively (Figure 4G, Additional file 1: Table S1B, 5A).

The enrichment of AP-1 motifs in GR peaks occurs primarily in pre-programmed GR peaks. 66% of the pre-programmed GR peaks (3,891/5,865) overlapped with c-Jun peaks, 13% of the re-programmed GR peaks (259/1,946) overlapped with c-Jun peaks, and only 1% of un-programmed GR peaks (3/425) co-occur with a c-Jun peak (Figure 3A). When examined from the c-Jun perspective, 20% of the pre-programmed c-Jun peaks (3,812/19,370) have a GR peak while 93% of re-programmed c-Jun peaks co-occur with the GR peaks (297/321) suggesting a mechanism where c-Jun promotes GR binding by creating new DHSs, and only 13% of the un-programmed c-Jun peaks co-occur with a GR peak (91/700) (Figure 3B).

### Bound 1-bp variants of the GR motif are more enriched for AP-1 motifs than canonical GR motifs

Several GR-like split 8-mers are enriched in GR peaks and the 5 most enriched sequences are 1-bp variants of the canonical GR motif. The occurrence of the GR canonical motif and its 1-bp variants in the pre-, re-, and un-programmed GR peaks (Table 2, Additional file 1: Figure S7A) showed that the two canonical GR motifs and the 1-bp variants are more frequent in the re-programmed and un-programmed GR peaks than the pre-programmed GR peaks. For all 1-bp variants of GR motif in the genome, we determined what percent are bound by GR and if one or more AP-1 motifs was within 150-bps. Relaxing the GR motif of G-ACA---^T^/_C_GT-C by 1-bp decreased GR binding to less than 1% of all occurrences in the genome. However, the presence of AP-1 motifs within 150-bps to the 1-bp variants of GR motif increased GR binding by ~8-fold (Figure 4H). Taken together, AP-1 motifs contribute significantly to GR binding but preferentially to non-canonical GR motifs. Similar to the canonical AP-1 motif, 1-bp variants of the AP-1 motif can contribute to GR binding (Additional file 1: Table S1B and Table S5A-B). Co-occurrences of AP-1 motif with 1-bp variants of GR motifs are always higher than those with the canonical GR motifs in the GR ChIP-seq peaks, although their binding intensity is lower than the canonical GR motifs (Table 2, Additional file 1: Figure S7B). These data suggest a prominent role of AP-1 in creating DHS that subsequently facilitate GR binding to 1-bp GR variants in the pre-programmed DHS [22].

### Co-occurring AP-1 motifs need c-Jun binding for GR binding

The contribution of c-Jun binding to GR binding was examined using A-FOS, a dominant negative protein that heterodimerizes with c-Jun and prevents DNA binding [22,33]. The GR peaks with more than a 50% decrease in tag-density upon A-FOS expression are considered c-Jun dependent. For the pre-programmed motifs, A-FOS expression inhibited 44% of GR binding to canonical GR motifs when there was an AP-1 motif within 150-bps but only 15% of GR binding to canonical GR motifs without a nearby canonical AP-1 motif. Similar results were observed for all 1-bp variants of GR motifs (Table 2, Additional file 1: Figure S8). A-FOS expression had similar effects on the re- and un-programmed GR peaks that contain a GR and AP-1 motif within 150-bps. The effect of A-FOS was lower in the re- and un-programmed GR peaks without an AP-1 motif again supporting the observation that presence of nearby AP-1 motif helps create a DHS facilitating GR binding to the canonical and 1-bp variants of GR motifs.

### GR binds different GR motifs in AtT-20 cells

**Figure 5:**
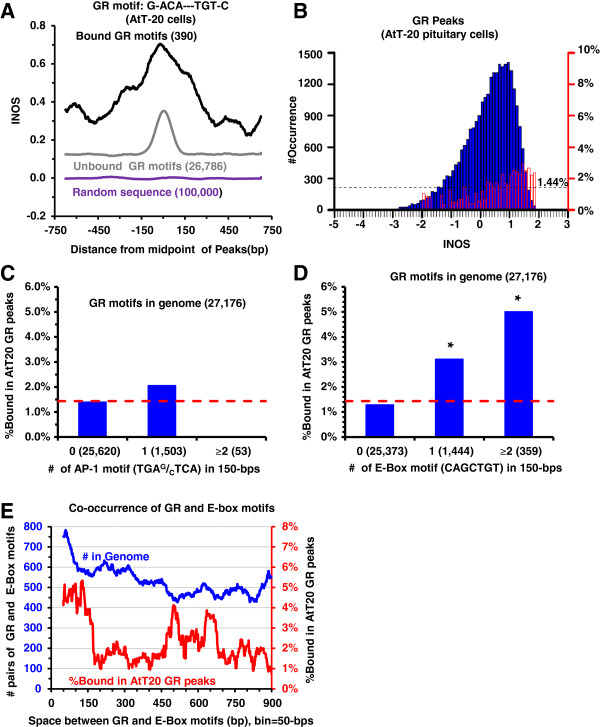
**In AtT**-**20 pituitary cells**, **GR binds regions with high INOS and co**-**occurs with E**-**box motifs.** (**A**) Average INOS near (±750-bps) canonical GR motifs (G-ACA---TGT-C); 390 bound and 26,786 unbound GR motifs in AtT-20 pituitary cells are shown. (**B**) GR binds preferentially to the GR motifs with higher INOS in AtT-20 cell line. See Figure 1C. 1.44% of all GR sites are bound by GR (black dashed line). (**C**) AP-1 motifs are not enriched in GR peaks in AtT-20 cells. (**D**) Number of GR motifs with 0, 1, or 2 or more E-box 7-mer (CAGCTGT) within 150-bps in the genome and percent bound by GR are presented along y-axis. The * denotes the statistical significance (p<0.01) of co-occurring GR and E-Box motifs being bound compared to GR motifs without an E-Box motif. The red dashed line in panels **C**-**D** is the average binding ratio of the GR motif. (**E**) The co-occurrence (9,651) of a canonical GR and E-Box motif at various distances up to 900-bps in the masked mouse genome and fraction in AtT-20 GR ChIP-seq peaks. The bin size used is 50-bps with 1-bp sliding window.

In a mouse pituitary AtT-20 cell line, GR bound different canonical GR motifs that also have high INOS (Figure 5A-B), suggesting that this trait is a general principle regulating GR localization (Additional file 1: Table S1B). The logistic regression analysis indicates that the PVE and AUC of INOS in peaks for GR localization are 2.6% and 0.65 in AtT-20 cells (Additional file 1: Table S1B). Instead of co-occurrence with AP-1 motifs (Figure 5C), GR co-occurred with the E-Box motif (CAGCTGT) in AtT-20 cells (Figure 5C-E, Additional file 1: Table S5C) suggesting that the co-occurrence of a second TFBS might be a cell type dependent mechanism. The addition of nearby E-box motif to the logistic regression increased the PVE for GR binding from 2.6% to 3.5% (Additional file 1: Table S1B).

## Discussion

The advent of the ChIP-seq technique [34-36] has produced detailed maps of transcription factor binding in the genome. These data sets are often used to produce a position weight matrix for preferred binding sites. However, it is difficult to compare the properties of one TF with another. We have used a logistic regression to evaluate sequence properties near TFBS that are predicative of a TFBS being bound *in vivo*. We examined the INOS of TFBS embedded in 150-bps of DNA and the co-occurrence of a secondary motif. These descriptors are different for the three TFs examined, GR, c-Jun, and Hoxa2 binding the GR, AP-1 and Pbx motifs respectively.

Recent computational models for calculating the strength of the *in vitro* binding of a nucleosome to any 150-bp length of DNA [4,5,37] allows evaluation of the significance of this parameter to TF localization in the genome. Many TFs bind in regions that have higher INOSs than neighboring DNA and are depleted for nucleosomes [5][30] suggesting a competition model of gene regulation [18]. We determined the INOS for each canonical GR, AP-1, and Pbx motif in the genome and identified which ones were bound by examining ChIP-seq data for GR and c-Jun. Our analyses showed that both GR and c-Jun bind preferentially to the canonical motifs predicted to be well bound by nucleosomes (Figure 1) lending support to the competition model of gene regulation [18] that we were able to quantify using a logistic regression. These data indicate that high INOS is more predictive of c-Jun localization (PVE = 11.5, and AUC = 0.77) than GR localization (PVE = 3.9, and AUC = 0.66) with Pbx showing little localization dependent on high INOS (PVE = 0.6, and AUC = 0.57). The greater value for c-Jun localization to AP-1 motifs than GR binding to GR motifs suggest c-Jun may be more involved in competitive interactions with nucleosomes, such as maintaining or creating DHS [22]. Both GR and Pbx binding motifs with low INOS suggesting a second mode of function for these two proteins. The significance of these differences observed among GR, c-Jun and Hoxa2 using the logistic regression will become clearer as additional TFs are examined. More accurate models of sequence specific nucleosome binding will improve the accuracy of these predictions.

Competition models for TF and nucleosome binding the same sequence have been extended to collaborative competition models where two TFs can bind to DNA independently but on their own do not displace a nucleosome. However, cooperatively they displace a nucleosome if they are within 150-bps [10,11,15-18]. This switch mechanism is observed in mammalian genomes [3,7] where regulatory regions [1] are often CpG islands that are well bound by nucleosomes *in vitro*[8] but not *in vivo*. The mechanism for the switch is complex. For example, some TFs (e.g. GR) can bind to a motif that is also bound by the histone octamer, while other TFs (e.g. c-Jun) can only bind to motif only when the histone octamer is not bound. Additional cooperativity to the switch can be achieved with cooperative binding of TF to their TFBSs [28,38].

The ability of two TFs to compete with a nucleosome for binding suggests that they are within 150-bps of each other [18]. When we examine the entire genome, there are ~1,500 occurrences of GR and AP-1 motifs within 150-bps, compared to ~1,100 occurrences expected, indicating that co-occurring GR and AP-1 motifs are enriched in the genome. In the mammalian genome, the clustering of TFBS is always observed when the two TFBS contain a CG dinucleotide [9] because of the presence of CpG islands in the genome. However, neither the abundant GR motif (G-ACA---TGT-C) nor the AP-1 motif contains a CG dinucleotide suggesting their preferential localization in the genome is independent from the clustering of TFBS containing CG. For the rare CG dinucleotide containing GR motif (G-ACA---CGT-C), the methylation status of the CG can regulate function adding a layer of complexity onto GR activity [39].

The collaborative competition model allows a single TF to interact with different TF in different cell types producing multiple functions. This is consistent with the observations concerning GR co-localization with different DNA motifs in the two cell types examined. In the mouse pituitary cell line AtT-20, GR binds GR motifs that have high INOS. But instead of being enriched in AP-1 motifs, GR binds canonical motifs enriched in the co-localizing E-Box motif that are within 150-bps. GR binds preferentially to motifs with high INOS in both 3134 and AtT-20 cells. Thus, the propensity to bind a nucleosome may be a general parameter in determining GR localization. However, the nucleosome may be displaced by different collaborative TFs in difference cells, suggest that nucleosome positioning can be cell-specific.

TFBS are often clustered in regulatory regions [18,32,40,41] where low-affinity TFBSs may play a role in assisting high-affinity motifs bind a TF(s) to displace a nucleosome [11,42]. 83% of GR peaks and 42% of c-Jun peaks do not contain canonical motifs. However, many 1-bp variants are enriched in GR and c-Jun peaks, allowing us to survey the difference of collaborative competition for canonical and 1-bp variants. We observed that a canonical AP-1 motif facilitates GR binding to 1-bp variants (~8 folds) better than the two canonical GR motifs (~3 folds).

The intrinsic nucleosome occupancy is correlated with GC content, which implies that some TFBSs with high GC content may be well bound by nucleosomes [4,5,8,37], while others with low GC content may be not. Both GR and AP-1 canonical motifs are calculated to be well bound by nucleosomes. The GR (G-A**CA**---**TG**T-C) and AP-1 (**TG**A^C^/_G_T**CA**) motifs have two pyrimidine-purine dinucleotides (in bold) separated by 5-bps. These sequences wrap well around a nucleosome, which may be the reason why nucleosomes are calculated to bind these sequences well [43]. Besides GR and AP-1 motifs, many additional 8-mers, such as the CRE (TGACGTCA) and CTCF motif (AGGGGGCG) have a high INOS, which indicates that many TFs bind to the same sequences calculated to be well-bound by the nucleosome and produce an intrinsic competition between TF and nucleosomes for binding the same DNA. In contrast, some motifs, e.g., Pbx (TGATTGAT) bound by Hoxa2 are not well bound by nucleosomes suggesting they are not competing for binding to the same DNA. Hoxa2 ChIP-seq data showed binding to motifs with both high and low INOS suggesting that this protein can function in two separate mechanisms. These results are consistent with what Charoensawan et. al recently observed in yeast [29].

Examples of a non-competitive model for TF and nucleosome binding are observed in yeast. Generally, yeast promoters are AT-rich with lower nucleosome occupancy both *in vitro* and *in vivo*[4,44-46]. A recent study showed that, in yeast the transcriptional activators with high intrinsic nucleosome binding properties might compete with nucleosomes, while the repressors are intrinsically less likely to compete with nucleosomes [29]. In yeast, TFs can recognize a specific regulatory region in the genome background to regulate gene expression, while in higher eukaryotes, such as human and mouse, TFBSs must be clustered to achieve specificity and collaborate to compete with nucleosomes [2].

## Conclusion

We have used a logistic regression to quantify the contribution of INOS and co-occurring sequence to TF binding in the genome. This strategy will allow investigators to more richly compare the properties of different TFs. Only AP-1 motifs with high INOS were preferentially bound while GR and Hoxa2 bound canonical motifs with both high and low INOS suggesting these proteins can function using two mechanisms.

## Methods

### Data sets

The reference genome of mouse (masked and unmasked, mm9) and DHS peaks in 55 samples from ENCODE project [47] are obtained from University of California Santa Cruz Genome Bioinformatics website (http://genome.ucsc.edu/) [48,49]. The data of GR ChIP-seq peaks, DHS peaks and expression array data in mouse 3134 mammary cells and pituitary cell line AtT-20 is from previous study [21] deposited at NCBI with SRA number of SRP004871 and GEO number of GSE26189. The data of c-Jun ChIP-seq peaks and GR ChIP-seq peaks upon A-FOS is from the study [22] deposited at NCBI with SRA number of SRP007111. The data of Hoxa2 ChIP-seq peaks is downloaded from supplementary data of Donaldson et. al’s study [23] deposited at NAR Online. The canonical motifs for GR, AP-1 and Pbx are selected based on the enrichment of GR-like 8-mers, 7-mers, 8-mers in GR, c-Jun and Hoxa2 ChIP-seq peaks. Custom Perl scripts are used to search GR, AP-1 and Pbx motifs base by base across the whole masked mouse genome of mm9, and to extract the sequences with 750-bps upstream and downstream to the center of each peak and motif from the unmasked genome of mm9.

### Enrichment of GR-like 8-mers and AP-1 like 8-mers in ChIP-seq peaks

To calculate the enrichment of different 8-mers, we first generated the unique 32,896 8-mers by ignoring the complementary reverse 8-mers. Then we extracted the sequences with 750-bps upstream and downstream to the center of each ChIP-seq peak from the unmasked genome of mm9. For each sequence, we defined the DNA fragment of ±150-bps to the center of peak as Peak region, and ±750-bps to ±150-bps to the center of peak as Background region. For each 8-mer, we count the occurrence of the 8-mer in Peak region as #PK, and occurrence of the 8-mer in Background region as #BG. The enrichment for each 8-mer (E_8-mer_) is then calculated as: $E_{8-\mathrm{mer}}=\frac{\#\mathrm{PK}}{\#\mathrm{BG}}\;\times \;\left(\frac{1500-300}{300}\right)$.

### Intrinsic nucleosome occupancy calculation

Two models for calculation of intrinsic nucleosome occupancy are used in our analysis. One is intrinsic nucleosome occupancy score (INOS) based on Lasso algorithm from Hughes’ group [5,37] and the second model to predict nucleosome occupancy probability (PNOP) uses Segal’s model [4]. For each 1,500-bps sequence, we calculated the INOSs and PNOPs for each 147-bps slide window and moved the window one base-pair at a time to get the profile of INOSs and PNOPs. The control set is 100,000 sequences random selected from mm9. The Peak value of INOS is calculated from the middle 147-bps of each peak or motif. The Background value of INOS is the average INOSs of regions from ±750 to ±150-bps to the peak or motif. The Relative Peak value of INOS is calculated as Peak value minus Background value. The INOS for excluding a specific motif (G-ACA---TGT-C or TGA^C^/_G_TCA) is calculated from the sequence whose motif is replaced by random bases but with GC content of 42% as in the mouse genome.

### Modeling GR and c-Jun binding by a logistic regression

To analyze the GR and c-Jun binding to the canonical motifs, we performed a logistic regression using the generalized linear model (GLM) with the R statistical language. GLMs were formulated by John Nelder and Robert Wedderburn as a way of unifying various other statistical models, including linear regression, logistic regression and Poisson regression [50]. GLM is a standard package in R language for computation and modeling. For each motif M_i_, the binding value (BV_i_) for M_i_ is 1 if motif M_i_ occurs in the ChIP-seq peaks, otherwise BV_i_ is 0. Three INOSs for each M_i_ are used for evaluated parameters: INOS of the Peak denoted as INOS^p^, INOS of the Backgroud denoted as INOS^b^, INOS of the Relative Peaks denoted as INOS^rp^. For the parameters of overlap with CGIs (CGI), with in clusters (CLT), co-occurrence with the second motifs (CO), and located in DHSs (DHS), if it is true, the value is 1, otherwise is 0. The formula for calculated the GLM in R is: BV~ INOS^p^ + INOS^b^ + INOS^rp^ + CGI + CLT + CO + DHS, with the binomial distribution. Let P_BV_ be the conditional probability of motif M_i_ being bound, which is generated from the independent variables of INOS^p^, INOS^b^,INOS^rp^, CGI, CLT,CO, and DHS: P_BV_ = P{BV=1| INOS^p^, INOS^b^, INOS^rp^, CGI, CLT, CO, DHS}, then the logistic regression is:

(1) $$P_{\mathrm{BV}}=\frac{\;\text{exp}\;\left(\beta_{0}+\beta_{1}\times {\text{INOS}}^{p}+\beta_{2}\times {\text{INOS}}^{b}+\beta_{3}\times {\text{INOS}}^{\mathrm{rp}}+\beta_{4}\times \mathrm{CGI}+\beta_{5}\times \mathrm{CLT}+\beta_{6}\times \mathrm{CO}+\beta_{7}\times \text{DHS}\right)}{1+\;\text{exp}\;\left(\beta_{0}+\beta_{1}\times {\text{INOS}}^{p}+\beta_{2}\times {\text{INOS}}^{b}+\beta_{3}\times {\text{INOS}}^{\mathrm{rp}}+\beta_{4}\times \mathrm{CGI}+\beta_{5}\times \mathrm{CLT}+\beta_{6}\times \mathrm{CO}+\beta_{7}\times \text{DHS}\right)}$$

Where β_0_ is constant, and β_1_, β_2_, β_3_, β_4_, β_5_, β_6_, and β_7_ are the coefficients for INOS^p^, INOS^b^, INOS^rp^, CGI, CLT, CO, and DHS respectively. By logit transformation (link function), a linear regression is generalized from formula (1), as follows:

(2) $$\begin{array}{l} \mathrm{logit}\left(P_{\mathrm{BV}}\right)=\ln\left[P_{\mathrm{BV}}/\left(1-P_{\mathrm{BV}}\right)\right]\\ \;=\beta_{0}{+\beta }_{1}\times {\text{INOS}}^{p}+\beta_{2}\times {\text{INOS}}^{b}+\beta_{3}\\ \;\times {\text{INOS}}^{\mathrm{rp}}+\beta_{4}\times \mathrm{CGI}+\beta_{5}\times \mathrm{CLT}\\ \;+\beta_{6}\times \mathrm{CO}+\beta_{7}\times \text{DHS} \end{array}$$

The percent of variance explained (PVE) is calculated as: PVE = (1-(deviance/null.deviance)) × 100. For each parameter, the PVE denotes the significance for predicting GR or c-Jun binding: the higher value of PVE means the parameter is more predictive. We also estimated area under the ROC curve (AUC) using 11-fold cross-validation to measure the predictive ability of the logistic regression model for comparison between TFs.

### Calculation of INOS for GR-like 8-mers and AP-1-like 8-mers

To calculate the INOS for each 8-mer, either GR-like (N-NNN---NNN-N) or AP-1-like (NNNNNNNN), we first simulated a 150-bps DNA sequence using Markov model with the 8-mer fixed in the center and with the same GC content (42%) as mouse genome. We used the seventh-order Markov model to produce the simulated 150 bps. The DNA sequences were generated by using the 8-mer frequencies observed in mouse genome. To populate each 150-bps DNA sequence, initially an 8-mer was chosen at random. To determine each next base, the preceding 7-mer was identified. The frequency of the four 8-mers starting with this 7-mer was determined, and the next base-pair was chosen by chance maintaining this frequency. This process was continued until the entire 150-bps sequence was determined. Then we calculated the INOS for the 150-bps DNA sequence. For each 8-mer, we repeat the simulation for 1,000 times and the average value of the 1,000 INOSs is treated as the INOS for the 8-mer. A random control set of DNA sequences are also calculated with all 150-bps are simulated using Markov model with GC content of 42%. For each control 8-mer, 1,000 sequences 150-bps long are simulated, and 32,896 times are repeated to get the whole random control set.

### Simulation of co-occurrence of GR and AP-1 canonical motifs

To simulate the co-occurrence of GR and AP-1 canonical motifs in the genome, we used uniform location model: the same occurrences of GR and AP-1 canonical motifs are generated in each masked chromosome, but each location of the motif is selected uniformly at random from each masked chromosome. For each chromosome with length N, we first generated the locations where bases are not Ns (A|C|T|G), as {X+1, X+2, X+n}, X∈1,…N. If the canonical motif occurs in the chromosome M times, then we random selected M positions from {X+1, X+2, … , X+n} as the simulated occurrence. Then we simulated for all the 22 chromosomes to get the whole genome simulation. After generating the simulated occurrence of GR and AP-1 canonical motifs, we calculated the distance between the two motifs. 1,000 same simulations are repeated to generate the distribution of co-occurrence of simulated GR and AP-1 motifs with 150-bps and from 150-bps to 900-bps. Similarly, we simulated co-occurrence of the GR and AP-1 motifs in the DHS regions (~10% genome), where all the simulated GR and AP-1 motifs occurred only in the DHS regions.

## Abbreviations

TF: Transcription factor; TFBS: Transcription factor binding site; GLM: Generalized linear model; INOS: Intrinsic nucleosome occupancy score; DHS: DNase I hypersensitive sites; PVE: Percent of variance explained; PNOP: Predicted nucleosome occupancy probability; GR: Glucocorticoid receptor; Hoxa2: Homeobox a2; Pbx: Pre-B-cell leukemia homeobox; AUC: Area under the ROC curve.

## Competing interests

The authors have no competing interests to declare.

## Authors’ contributions

XH, RC, BKS performed the computational analyses. SJ and SCB conducted the experiments. XH, HB, PCF, JAS, GLH and CV conceived of the study. XH drafted the manuscript. RC and CV helped to revise the manuscript. All authors read and approved the final manuscript.

## Supplementary Material

Additional file 1: Supplementary Figures and Tables. Click here for file

## References

[B1] FitzGerald PC Shlyakhtenko A Mir AA Vinson C Clustering of DNA sequences in human promoters Genome Res 2004 14 1562 1574 10.1101/gr.1953904 15256515 PMC509265

[B2] Wunderlich Z Mirny LA Different gene regulation strategies revealed by analysis of binding motifs Trends Genet 2009 25 434 440 10.1016/j.tig.2009.08.003 19815308 PMC3697852

[B3] FitzGerald PC Sturgill D Shyakhtenko A Oliver B Vinson C Comparative genomics of Drosophila and human core promoters Genome Biol 2006 7 R53 10.1186/gb-2006-7-7-r53 16827941 PMC1779564

[B4] Kaplan N Moore IK Fondufe-Mittendorf Y Gossett AJ Tillo D Field Y LeProust EM Hughes TR Lieb JD Widom J Segal E The DNA-encoded nucleosome organization of a eukaryotic genome Nature 2009 458 362 366 10.1038/nature07667 19092803 PMC2658732

[B5] Tillo D Kaplan N Moore IK Fondufe-Mittendorf Y Gossett AJ Field Y Lieb JD Widom J Segal E Hughes TR High nucleosome occupancy is encoded at human regulatory sequences PLoS One 2010 5 e9129 10.1371/journal.pone.0009129 20161746 PMC2817738

[B6] Kaplan N Hughes TR Lieb JD Widom J Segal E Contribution of histone sequence preferences to nucleosome organization: proposed definitions and methodology Genome Biol 2010 11 140 10.1186/gb-2010-11-11-140 21118582 PMC3156944

[B7] Vinson C Chatterjee R Fitzgerald P Transcription factor binding sites and other features in human and Drosophila proximal promoters Subcell Biochem 2011 52 205 222 10.1007/978-90-481-9069-0_10 21557085 PMC7394279

[B8] Valouev A Johnson SM Boyd SD Smith CL Fire AZ Sidow A Determinants of nucleosome organization in primary human cells Nature 2011 474 516 520 10.1038/nature10002 21602827 PMC3212987

[B9] Rozenberg JM Shlyakhtenko A Glass K Rishi V Myakishev MV FitzGerald PC Vinson C All and only CpG containing sequences are enriched in promoters abundantly bound by RNA polymerase II in multiple tissues BMC Genomics 2008 9 67 10.1186/1471-2164-9-67 18252004 PMC2267717

[B10] Polach KJ Widom J Mechanism of protein access to specific DNA sequences in chromatin: a dynamic equilibrium model for gene regulation J Mol Biol 1995 254 130 149 10.1006/jmbi.1995.0606 7490738

[B11] Mirny LA Nucleosome-mediated cooperativity between transcription factors Proc Natl Acad Sci USA 2010 107 22534 22539 10.1073/pnas.0913805107 21149679 PMC3012490

[B12] Bai L Ondracka A Cross FR Multiple sequence-specific factors generate the nucleosome-depleted region on CLN2 promoter Mol Cell 2011 42 465 476 10.1016/j.molcel.2011.03.028 21596311 PMC3119483

[B13] Richard-Foy H Hager GL Sequence-specific positioning of nucleosomes over the steroid-inducible MMTV promoter EMBO J 1987 6 2321 2328 2822386 10.1002/j.1460-2075.1987.tb02507.x PMC553635

[B14] Fedor MJ Lue NF Kornberg RD Statistical positioning of nucleosomes by specific protein-binding to an upstream activating sequence in yeast J Mol Biol 1988 204 109 127 10.1016/0022-2836(88)90603-1 3063825

[B15] Taylor IC Workman JL Schuetz TJ Kingston RE Facilitated binding of GAL4 and heat shock factor to nucleosomal templates: differential function of DNA-binding domains Genes Dev 1991 5 1285 1298 10.1101/gad.5.7.1285 2065977

[B16] Adams CC Workman JL Binding of disparate transcriptional activators to nucleosomal DNA is inherently cooperative Mol Cell Biol 1995 15 1405 1421 7862134 10.1128/mcb.15.3.1405 PMC230365

[B17] Polach KJ Widom J A model for the cooperative binding of eukaryotic regulatory proteins to nucleosomal target sites J Mol Biol 1996 258 800 812 10.1006/jmbi.1996.0288 8637011

[B18] Miller JA Widom J Collaborative competition mechanism for gene activation in vivo Mol Cell Biol 2003 23 1623 1632 10.1128/MCB.23.5.1623-1632.2003 12588982 PMC151720

[B19] Perlmann T Wrange O Specific glucocorticoid receptor binding to DNA reconstituted in a nucleosome EMBO J 1988 7 3073 3079 2846275 10.1002/j.1460-2075.1988.tb03172.x PMC454694

[B20] John S Sabo PJ Johnson TA Sung MH Biddie SC Lightman SL Voss TC Davis SR Meltzer PS Stamatoyannopoulos JA Hager GL Interaction of the glucocorticoid receptor with the chromatin landscape Mol Cell 2008 29 611 624 10.1016/j.molcel.2008.02.010 18342607

[B21] John S Sabo PJ Thurman RE Sung MH Biddie SC Johnson TA Hager GL Stamatoyannopoulos JA Chromatin accessibility pre-determines glucocorticoid receptor binding patterns Nat Genet 2011 43 264 268 10.1038/ng.759 21258342 PMC6386452

[B22] Biddie SC John S Sabo PJ Thurman RE Johnson TA Schiltz RL Miranda TB Sung MH Trump S Lightman SL Transcription factor AP1 potentiates chromatin accessibility and glucocorticoid receptor binding Mol Cell 2011 43 145 155 10.1016/j.molcel.2011.06.016 21726817 PMC3138120

[B23] Donaldson IJ Amin S Hensman JJ Kutejova E Rattray M Lawrence N Hayes A Ward CM Bobola N Genome-wide occupancy links Hoxa2 to Wnt-beta-catenin signaling in mouse embryonic development Nucleic Acids Res 2012 40 3990 4001 10.1093/nar/gkr1240 22223247 PMC3351182

[B24] Sekinger EA Moqtaderi Z Struhl K Intrinsic histone-DNA interactions and low nucleosome density are important for preferential accessibility of promoter regions in yeast Mol Cell 2005 18 735 748 10.1016/j.molcel.2005.05.003 15949447

[B25] Bailey TL Boden M Buske FA Frith M Grant CE Clementi L Ren J Li WW Noble WS MEME SUITE: tools for motif discovery and searching Nucleic Acids Res 2009 37 W202 W208 10.1093/nar/gkp335 19458158 PMC2703892

[B26] Jurka J Repbase update: a database and an electronic journal of repetitive elements Trends Genet 2000 16 418 420 10.1016/S0168-9525(00)02093-X 10973072

[B27] Meijsing SH Pufall MA So AY Bates DL Chen L Yamamoto KR DNA binding site sequence directs glucocorticoid receptor structure and activity Science 2009 324 407 410 10.1126/science.1164265 19372434 PMC2777810

[B28] Chatterjee R Zhao J He X Shlyakhtenko A Mann I Waterfall JJ Meltzer P Sathyanarayana BK Fitzgerald PC Vinson C Overlapping ETS and CRE Motifs ((G)/(C)CGGAAGTGACGTCA) Preferentially Bound by GABPalpha and CREB Proteins G3 (Bethesda) 2012 2 1243 1256 2012 23050235 10.1534/g3.112.004002 PMC3464117

[B29] Charoensawan V Janga SC Bulyk ML Babu MM Teichmann SA DNA sequence preferences of transcriptional activators correlate more strongly than repressors with nucleosomes Mol Cell 2012 47 183 192 10.1016/j.molcel.2012.06.028 22841002 PMC3566590

[B30] Lidor Nili E Field Y Lubling Y Widom J Oren M Segal E p53 binds preferentially to genomic regions with high DNA-encoded nucleosome occupancy Genome Res 2010 20 1361 1368 10.1101/gr.103945.109 20716666 PMC2945185

[B31] Pettersen EF Goddard TD Huang CC Couch GS Greenblatt DM Meng EC Ferrin TE UCSF Chimera–a visualization system for exploratory research and analysis J Comput Chem 2004 25 1605 1612 10.1002/jcc.20084 15264254

[B32] Gotea V Visel A Westlund JM Nobrega MA Pennacchio LA Ovcharenko I Homotypic clusters of transcription factor binding sites are a key component of human promoters and enhancers Genome Res 2010 20 565 577 10.1101/gr.104471.109 20363979 PMC2860159

[B33] Olive M Krylov D Echlin DR Gardner K Taparowsky E Vinson C A dominant negative to activation protein-1 (AP1) that abolishes DNA binding and inhibits oncogenesis J Biol Chem 1997 272 18586 18594 10.1074/jbc.272.30.18586 9228025

[B34] Valouev A Johnson DS Sundquist A Medina C Anton E Batzoglou S Myers RM Sidow A Genome-wide analysis of transcription factor binding sites based on ChIP-Seq data Nat Methods 2008 5 829 834 10.1038/nmeth.1246 19160518 PMC2917543

[B35] Kharchenko PV Tolstorukov MY Park PJ Design and analysis of ChIP-seq experiments for DNA-binding proteins Nat Biotechnol 2008 26 1351 1359 10.1038/nbt.1508 19029915 PMC2597701

[B36] Furey TS ChIP-seq and beyond: new and improved methodologies to detect and characterize protein-DNA interactions Nat Rev Genet 2012 13 840 852 10.1038/nrg3306 23090257 PMC3591838

[B37] Tillo D Hughes TR G+C content dominates intrinsic nucleosome occupancy BMC Bioinforma 2009 10 442 10.1186/1471-2105-10-442 PMC2808325 20028554

[B38] Martinez GJ Rao A **Immunology**. Cooperative transcription factor complexes in control Science 2012 338 891 892 10.1126/science.1231310 23161983 PMC3621126

[B39] Wiench M John S Baek S Johnson TA Sung MH Escobar T Simmons CA Pearce KH Biddie SC Sabo PJ DNA methylation status predicts cell type-specific enhancer activity EMBO J 2011 30 3028 3039 10.1038/emboj.2011.210 21701563 PMC3160184

[B40] Schroeder MD Pearce M Fak J Fan H Unnerstall U Emberly E Rajewsky N Siggia ED Gaul U Transcriptional control in the segmentation gene network of Drosophila PLoS Biol 2004 2 E271 10.1371/journal.pbio.0020271 15340490 PMC514885

[B41] Berman BP Pfeiffer BD Laverty TR Salzberg SL Rubin GM Eisen MB Celniker SE Computational identification of developmental enhancers: conservation and function of transcription factor binding-site clusters in Drosophila melanogaster and Drosophila pseudoobscura Genome Biol 2004 5 R61 10.1186/gb-2004-5-9-r61 15345045 PMC522868

[B42] Zhang C Xuan Z Otto S Hover JR McCorkle SR Mandel G Zhang MQ A clustering property of highly-degenerate transcription factor binding sites in the mammalian genome Nucleic Acids Res 2006 34 2238 2246 10.1093/nar/gkl248 16670430 PMC1456330

[B43] Sahu G Wang D Chen CB Zhurkin VB Harrington RE Appella E Hager GL Nagaich AK p53 binding to nucleosomal DNA depends on the rotational positioning of DNA response element J Biol Chem 2010 285 1321 1332 10.1074/jbc.M109.081182 19887449 PMC2801259

[B44] Ioshikhes IP Albert I Zanton SJ Pugh BF Nucleosome positions predicted through comparative genomics Nat Genet 2006 38 1210 1215 10.1038/ng1878 16964265

[B45] Field Y Kaplan N Fondufe-Mittendorf Y Moore IK Sharon E Lubling Y Widom J Segal E Distinct modes of regulation by chromatin encoded through nucleosome positioning signals PLoS Comput Biol 2008 4 e1000216 10.1371/journal.pcbi.1000216 18989395 PMC2570626

[B46] Tirosh I Barkai N Two strategies for gene regulation by promoter nucleosomes Genome Res 2008 18 1084 1091 10.1101/gr.076059.108 18448704 PMC2493397

[B47] Stamatoyannopoulos JA Snyder M Hardison R Ren B Gingeras T Gilbert DM Groudine M Bender M Kaul R Canfield T An encyclopedia of mouse DNA elements (Mouse ENCODE) Genome Biol 2012 13 418 22889292 10.1186/gb-2012-13-8-418 PMC3491367

[B48] Meyer LR Zweig AS Hinrichs AS Karolchik D Kuhn RM Wong M Sloan CA Rosenbloom KR Roe G Rhead B The UCSC Genome Browser database: extensions and updates 2013 Nucleic Acids Res 2013 41 D64 9 10.1093/nar/gks1048 23155063 PMC3531082

[B49] Kuhn RM Haussler D Kent WJ The UCSC genome browser and associated tools Brief Bioinform 2013 14 144 61 10.1093/bib/bbs038 22908213 PMC3603215

[B50] Nelder JA Wedderbu RW Generalized Linear Models Journal of the Royal Statistical Society Series a-General 1972 135 370 384 10.2307/2344614

