# Supplementary material for: Contribution of nucleosome binding preferences and co-occurring DNA sequences to transcription factor binding
Source: BMC Genomics. 2013 Jun 28;14:428. doi: 10.1186/1471-2164-14-428 (PMC3700821; doi:10.1186/1471-2164-14-428)
Supplement: Additional file 1: — Supplementary Figures and Tables. [file 1471-2164-14-428-S1.pdf]

Supplementary Figures:

Figure S1. Enrichment of 8-mers in GR, c-Jun and Hoxa2 ChIP-seq Peaks. (A) Enrichment of GR-like split 8-mers (N-NNN---NNN-N) and their occurrences in the GR ChIP-seq peaks. **(B)** Enrichment of AP-1-like 8-mers (NNNNNNNN) and their occurrences in the c-Jun ChIP-seq peaks. (**C)** Enrichment of 8-mers (NNNNNNNN) and their occurrences in the Hoxa2 ChIP-seq peaks.

Figure S2. Average intrinsic nucleosome occupancy score for canonical motifs. (A) Average INOS by Tillo's model [1-2] near (±750-bps) canonical GR motifs (G-ACA---TGT-C): GR ChIP-seq peaks contain 523 canonical motifs in pre-programmed DHS, 665 in re-programmed DHS, 205 without DHS, and unbound GR motifs. Fraction of peaks containing the canonical motif is shown in percent. (**B)** Average INOS by Tillo's model near (±750-bps) canonical AP-1 motifs (TGA$^{C}/_{G}$TCA): c-Jun ChIP-seq peaks contain 10,673 canonical motifs in pre-programmed DHS, 96 in re-programmed DHS, and 495 without DHS. The average INOS for 260,047 unbound AP-1 motifs is shown. 100,000 randomly selected sequences are shown. Fraction of peaks containing the canonical motif is shown as a percent (%). **(C)** Average intrinsic nucleosome occupancy probability by Segal's model [3] near (±750-bps) canonical GR motifs. (**D)** Average intrinsic nucleosome occupancy probability by Segal's model near (±750-bps) canonical AP-1 motifs. **(E)** Average INOS near (±750-bps) canonical GR motifs (G-ACA---TGT-C) with INOS from -2 to -1; 38 bound and 1,786 unbound GR motifs are shown.

Figure S3. (A) Average INOS near (±750-bps) the center of c-Jun CIP-seq peaks with and without AP-1motif (TGA$^{C}/_{G}$TCA). **(B)** Average INOS near (±750-bps) the center of GR CIP-seq peaks with and without GR motifs (G-ACA---TGT-C). **(C)** The histogram shows the distribution of INOS for all c-Jun ChIP-seq peaks with (blue)/without (red) AP-1 motif. **(D)** The histogram shows the distribution of INOS for all c-Jun ChIP-seq peaks with (blue)/without (red) GR motif.

Figure S4. Chromatin accessibility of GR and AP-1 motifs in 3134 cell line. (A) Number of sequencing reads within 150-bps for c-Jun ChIP-seq data at each of the AP-1 8-mers motifs (ATGAGTCA) in the genome compared to DHS reads. Motifs are labeled depending on their presence at pre-, re- and unprogrammed DHS. Unbound AP-1 motifs are presented in grey. **(B)** Number of sequencing reads within 150-bps for GR ChIP-seq at each of the GR motifs in the

genome compared to DHS reads. Sequence reads for GR and c-Jun ChIP-seq are normalized. **(C)** Density of sequenced tags for GR, c-Jun ChIP-seq, and DNase-seq were counted in 3-Kb up and downstream of the bound GR motifs. **(D)** Density of sequenced tags for GR, c-Jun ChIP-seq and DNase-seq were counted in 3-Kb up and downstream of the bound AP-1 motifs (ATGAGTCA). The bin size used is 300-bps with 150-bps sliding window.

Figure S5. Clusters of GR and AP-1 motifs are better bound. (A) The occurrence of clustered GR canonical motifs (G-ACA---TGT-C) in the genome and percent bound by GR are presented in different genomic scales from 150-bps to 100-Kbps. The black line denotes percent of GR motifs that are bound in GR peaks. **(B)** The occurrence of clustered AP-1 canonical motifs (TGA$^{C}/_{G}$TCA) and percent bound by c-Jun are presented in different genomic scales from 150-bps to 20-Kbps.

Figure S6. Co-occurrence of AP-1 motifs (TGA$^{C}/_{G}$TCA) within a nucleosomal distance promotes GR binding. (A) The occurrence of pairs of canonical GR and AP-1 motifs with space less than 150-bps in the GR ChIP-seq peaks. **(B)** GR binds preferentially to the GR motifs with co-occurrence of AP-1 motifs and with higher INOS. The blue solid histogram shows all 1,448 pairs of GR and AP-1 motifs with space less than 150-bps in genome, and the red blank histogram shows the binding ratio in GR peaks (average is 15.3%, black dashed line). **(C-D)** Enrichment of split 8-mers (N-NNN---NNN-N) vs. all continuous 8-mers in the **(C)** c-Jun and **(D)** Hoxa2 ChIP-seq peaks. Red dots represent the canonical GR motif and 1-bp variants, and the mismatched base is labeled in red. Blue dots represent the 8-mers with AP-1 motif.

Figure S7. The 1-bp variants of canonical GR motif need more co-occurrence of canonical AP-1 motifs to facilitate GR binding. (A) The relative occurrence of the canonical GR motif and 1-bp variants in pre-, re- and un-programmed GR peaks. The relative occurrence of motifs is calculated as the number of motifs bound per 1000 GR peaks. The motifs are ordered by the enrichment in GR peaks. **(B)** Correlation of tag-density of GR peaks and enrichment of the canonical GR motif and the other 12 1-bp variants in pre-programmed GR peaks. (**C-E**) Correlation of tag-density of GR peaks and co-occurrence of AP-1 motifs in **(C)** pre-programmed, (**D**) re-programmed and **(E)** un-programmed GR peaks with the canonical and 1-bp variants of GR motif. For each dot, the horizontal axis of tag-density is the average tag-density of GR peaks with this motif, and the vertical axis indicates the percentage of GR motifs with AP-1

motifs within 150 bps. The 118 canonical motifs and 3 variants (occurrence < 50) with AP-1 motifs nearby in pre-programmed GR peaks are labeled in each panel. **(F)** Enrichment of all GR-like (NN-NNN---NNN-NN) and AP-1-like (NNNNNNNNNN) 10-mers in the GR ChIP-seq peaks. The enrichment of each 10-mer is calculated as the normalized ratio of occurrence in GR peaks (±150 bps from the center of GR peaks) over occurrence in neighbors (±750 to ±150 from center of GR peaks). As expected, [G/A]G-ACA---TGT-C[C/T] and AG-ACA---CGT-C[C/T] are enriched, as high as nearly 40. A new pattern of AC-TAG---TGG-AC [gACtTAGggc] also shows a high enrichment.

Figure S8. Effect of A-FOS on GR binding to the canonical GR motif and 1-bp variants depends on the presence of an AP-1 motif within 150-bps. (A-C) Percent of GR motifs with (blue) /without (Red) an nearby AP-1 motif that loses GR binding after inhibition of c-Jun binding by expressing the dominant negative A-FOS) in (A) pre-programmed, **(B)** re-programmed and **(C)** un-programmed GR peaks.

References:

1. Tillo D, Hughes TR: **G+C content dominates intrinsic nucleosome occupancy.** *BMC Bioinformatics* 2009, **10:**442.
2. Tillo D, Kaplan N, Moore IK, Fondufe-Mittendorf Y, Gossett AJ, Field Y, Lieb JD, Widom J, Segal E, Hughes TR: **High nucleosome occupancy is encoded at human regulatory sequences.** *PLoS One*, **5:**e9129.
3. Kaplan N, Moore IK, Fondufe-Mittendorf Y, Gossett AJ, Tillo D, Field Y, LeProust EM, Hughes TR, Lieb JD, Widom J, Segal E: **The DNA-encoded nucleosome organization of a eukaryotic genome.** *Nature* 2009, **458:**362-366.

Figure S1. Enrichment of 8-mers in GR, c-Jun and Hoxa2 ChIP-seq Peaks

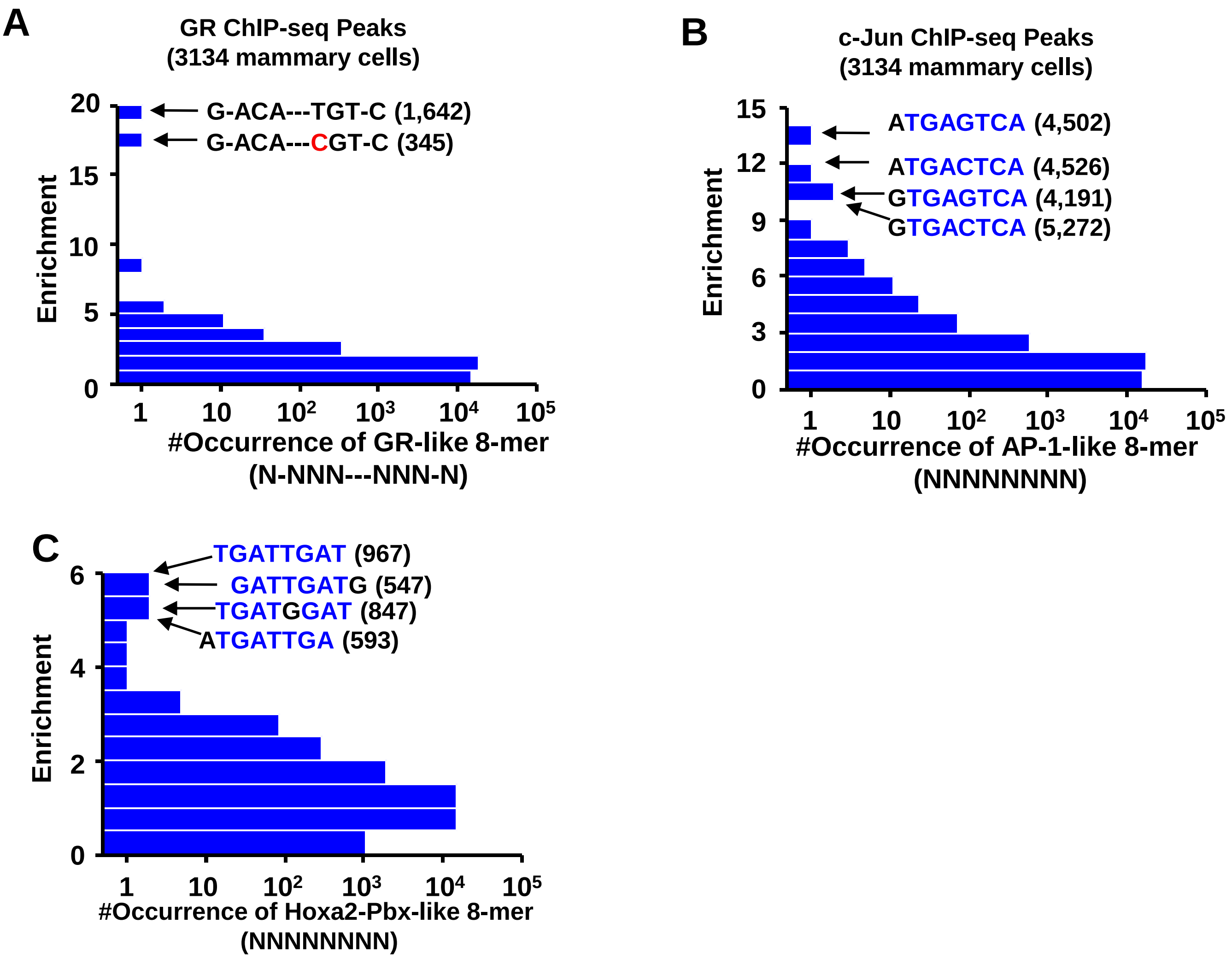

Figure S2. Nucleosome occupancy at regulatory sites

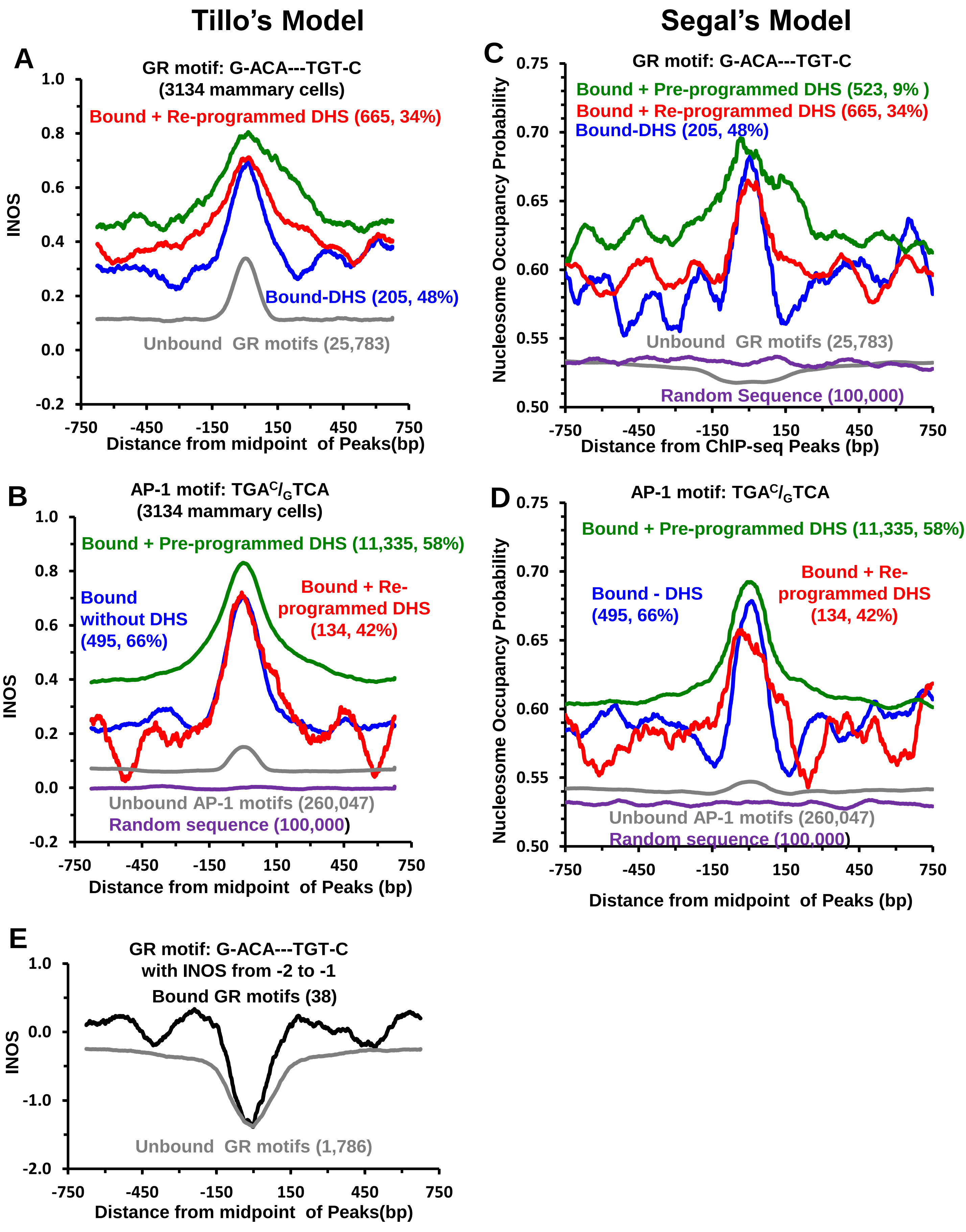

Figure S3. Nucleosome occupancy at regulatory sites

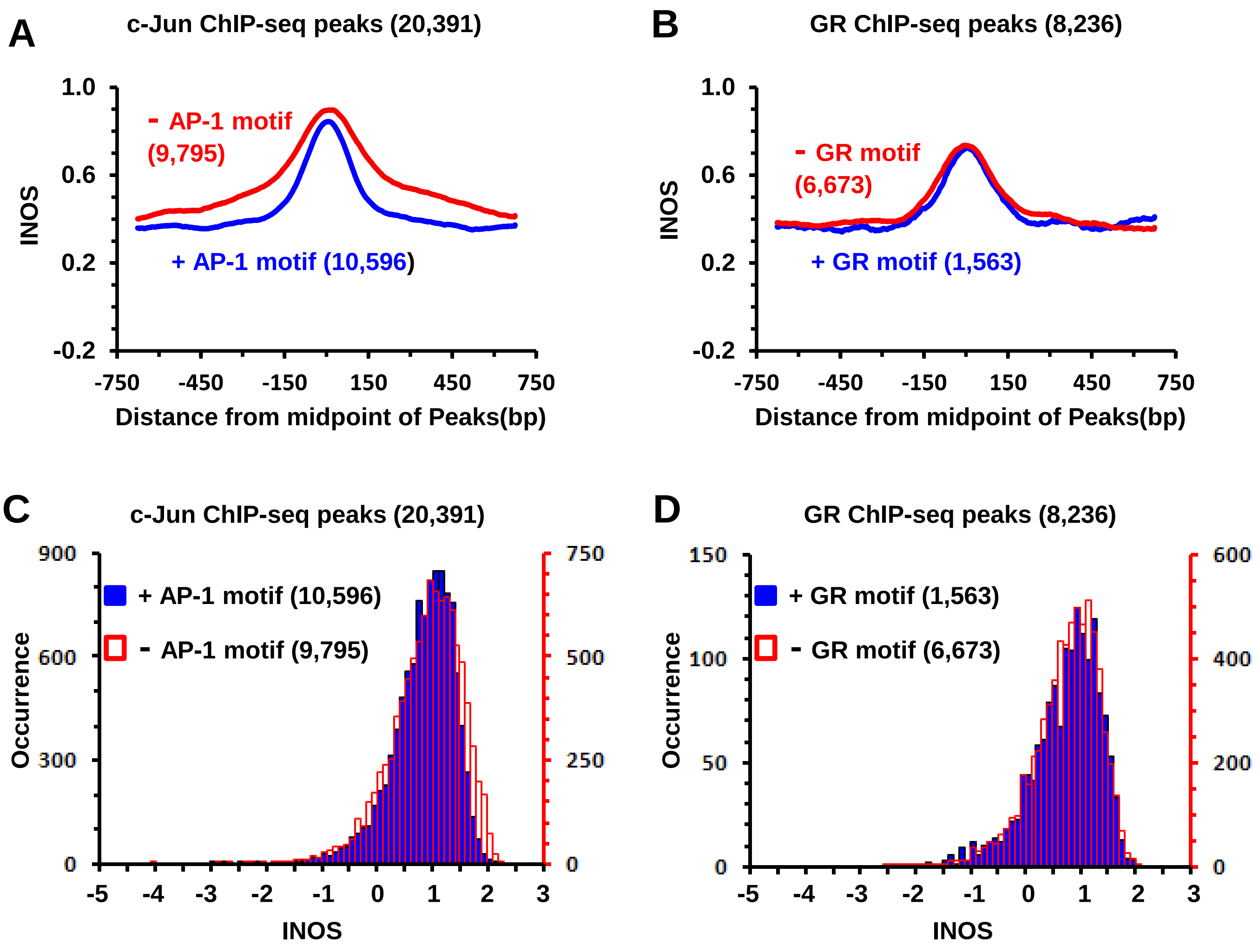

Figure S4. Chromatin accessibility of GR and AP-1 canonical motifs *in vivo*

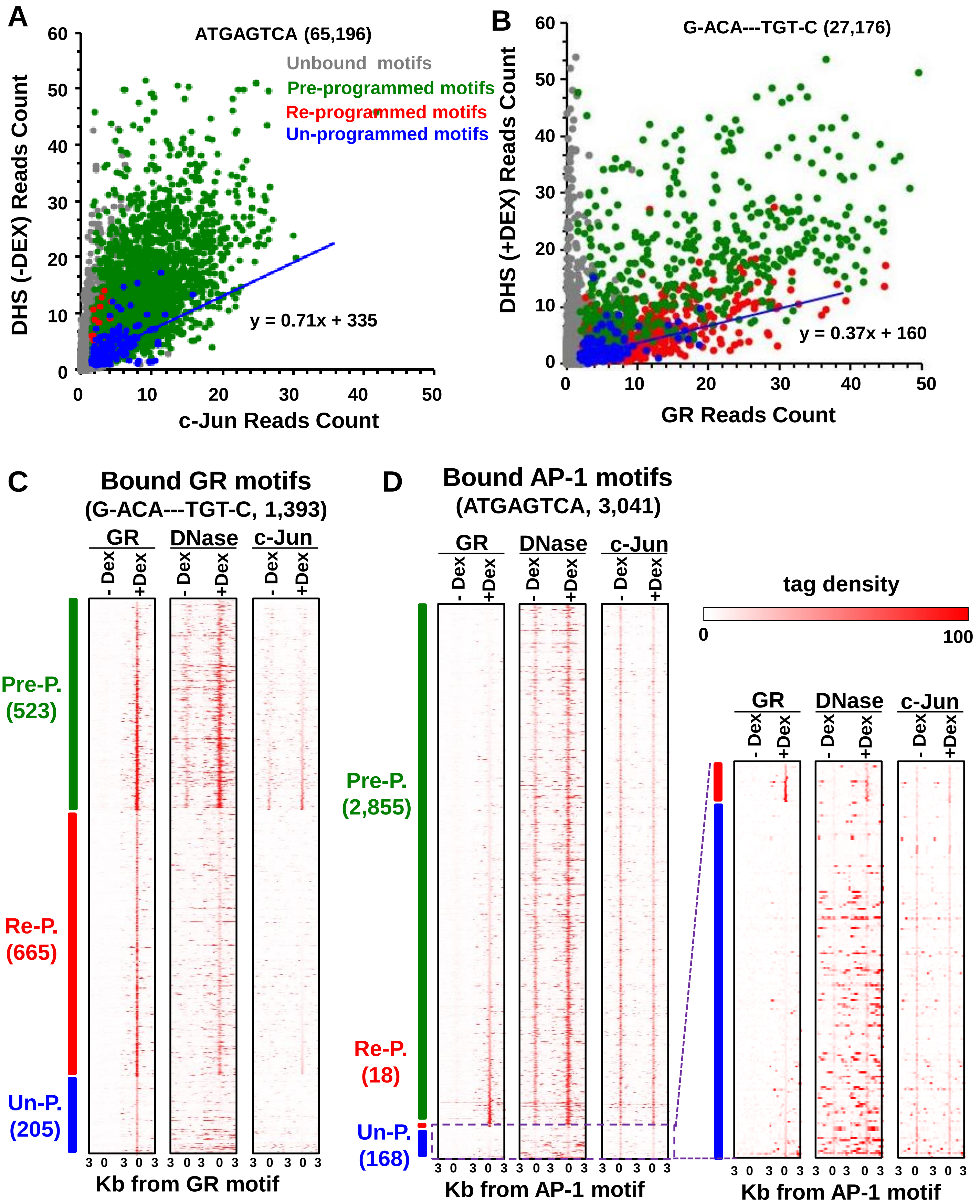

Figure S5. Clusters of GR and AP-1 motifs are better bound

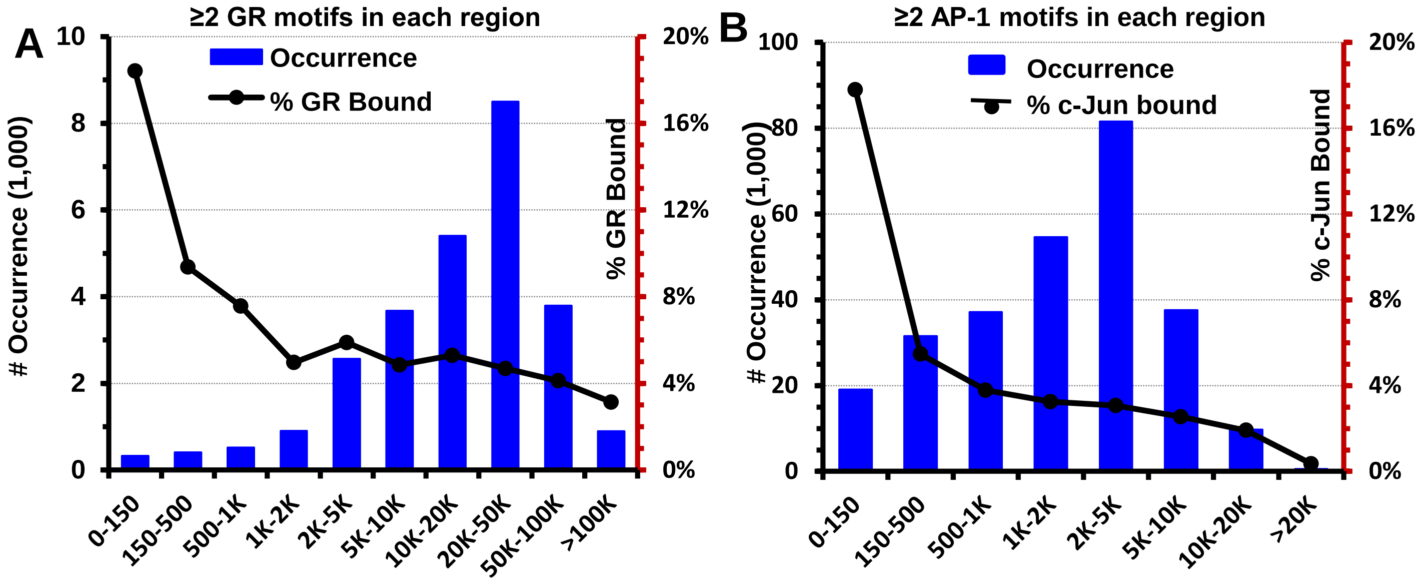

Figure S6. Co-occurrence of AP-1 motifs (TGA$^{C}/_{G}$TCA) within a nucleosome range promotes GR binding

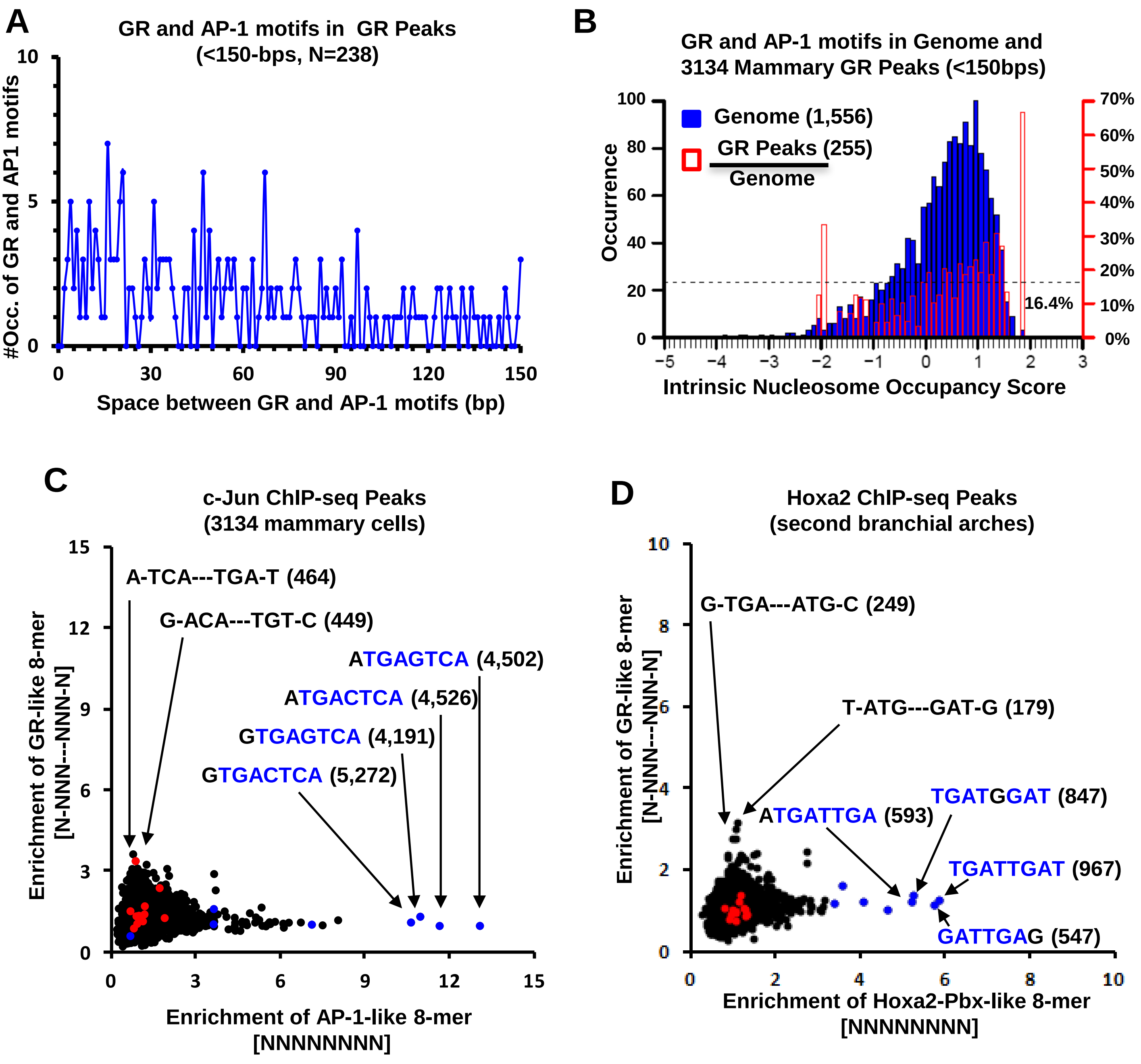

Figure S7. The 1-bp variants of GR motifs need more co-occurrence of AP-1 motifs to facilitate GR binding.

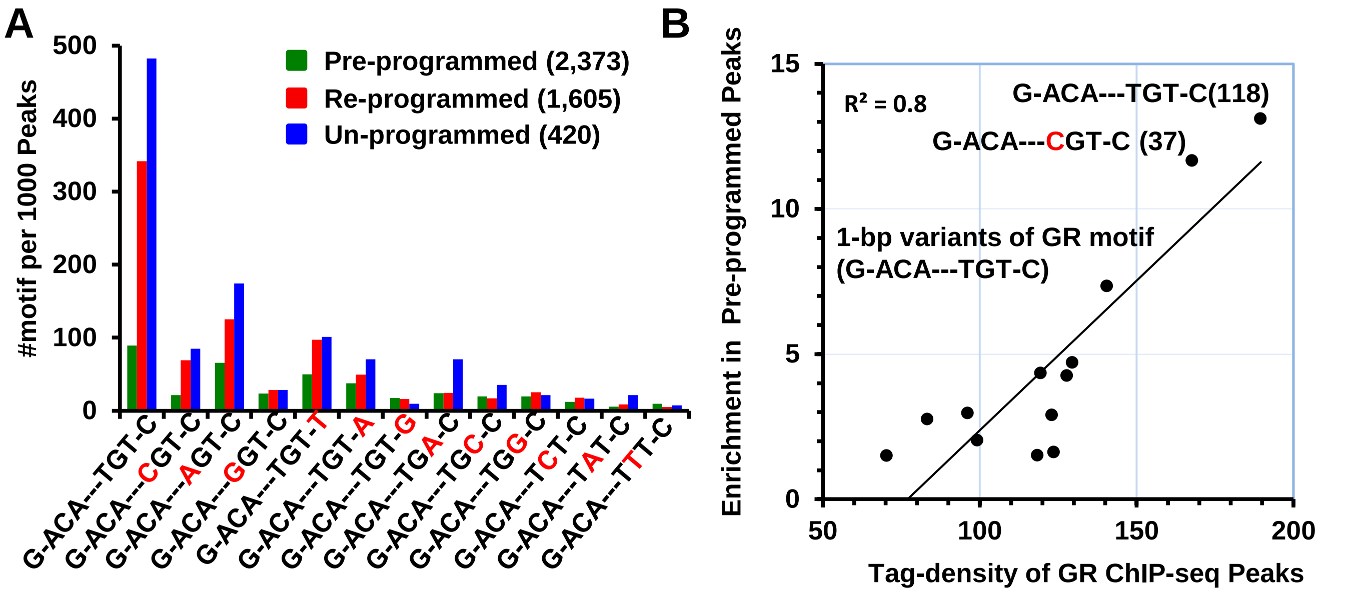

Figure S8. Effect of A-FOS on GR binding to the GR motif and 1-bp variants depends on the presence of an AP-1 motif within 150-bps.

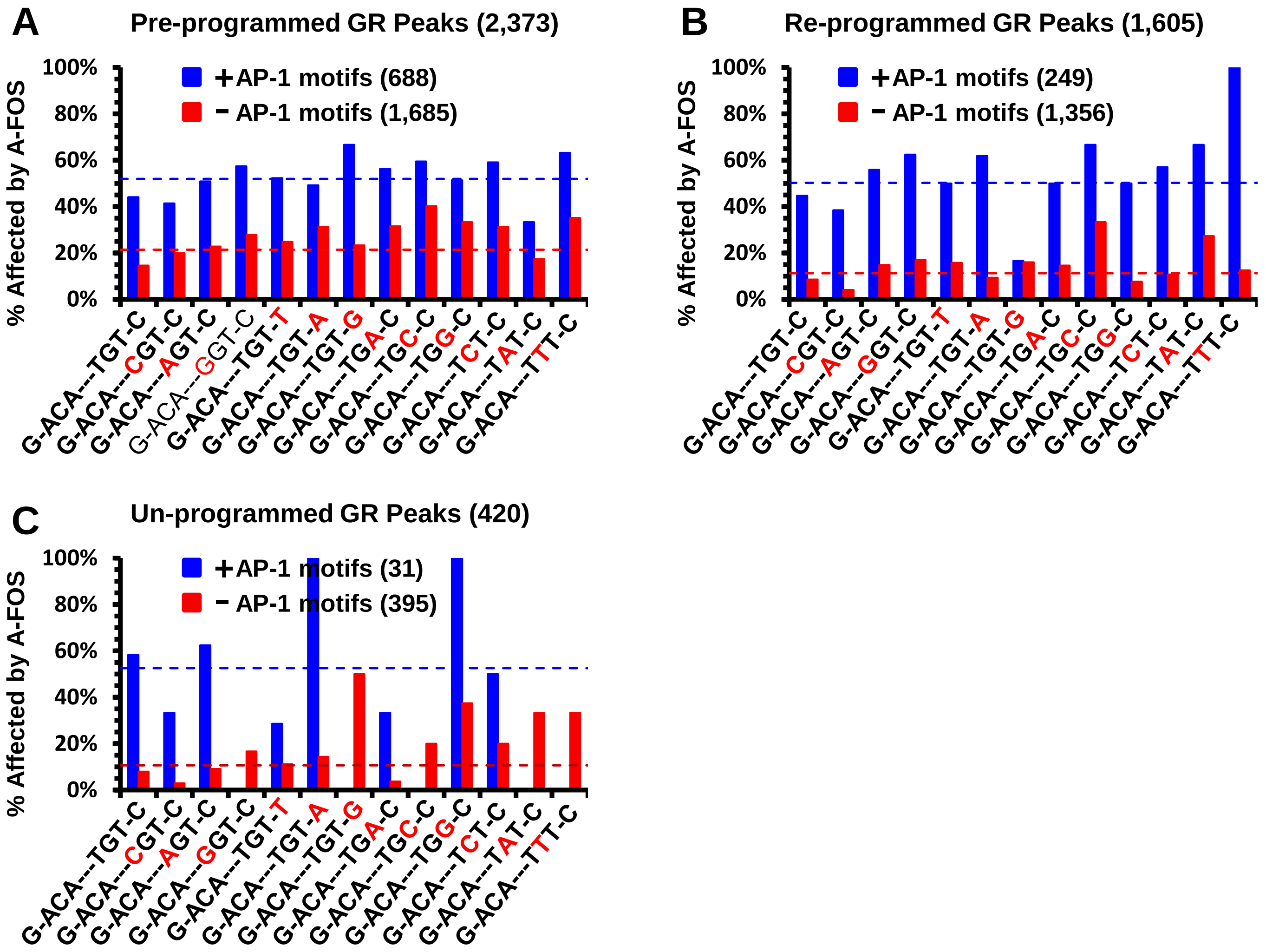

Supplementary Table 1A. Modeling the c-Jun binding of consensus AP-1 motif (TGACTCA|ATGAGTCA) using generalized linear models (GLM)

Evaluated parameters	ATGAGTCA +Motif PVE(%)	CV.err	AUC	ATGAGTCA −Motif PVE(%)	CV.err	AUC	TGAGTCA +Motif PVE (%)	CV.err	AUC	TGAGTCA −Motif PVE (%)	CV.err	AUC
**A1. Peak**	10.4	0.05	0.76	9.9	0.05	0.75	10.3	0.04	0.76	7.4	0.04	0.72
**A2. Background**	5.6	0.05	0.69	5.6	0.05	0.69	6.5	0.04	0.70	6.5	0.04	0.70
**A3. Relative peak**	2.5	0.05	0.63	2.3	0.05	0.63	1.9	0.04	0.62	1.6	0.04	0.61
**A. INOS (A1+A2+A3)**	**11.3**	**0.05**	**0.77**	**10.9**	**0.05**	**0.76**	**11.5**	**0.04**	**0.77**	**8.2**	**0.04**	**0.73**
**B. Overlap with CGIs**	0.8	0.05	0.53	0.8	0.05	0.53	0.9	0.04	0.53	0.9	0.04	0.53
**C. Cluster of AP-1 motifs**	0.8	0.05	0.53	-	-	-	5.5	0.04	0.66	-	-	-
**D. Co-occurrence of GR motifs**	**0.1**	**0.05**	**0.51**	**0.1**	**0.05**	**0.51**	**0.2**	**0.04**	**0.51**	**0.2**	**0.04**	**0.51**
**E. Chromatin Accessibility (DHS)**	55.1	0.05	0.94	55.1	0.05	0.94	56.5	0.04	0.93	56.5	0.04	0.93
**A+B**	11.4	0.05	0.77	11.1	0.05	0.77	12.3	0.04	0.78	8.3	0.04	0.73
**A+B+C**	12.2	0.05	0.78	11.9	0.05	0.77	13.9	0.04	0.79	13.5	0.04	0.78
**A+B+C+D**	12.3	0.05	0.78	12.0	0.05	0.77	14.0	0.04	0.79	13.6	0.04	0.78
**A+B+C+D+E**	56.1	0.04	0.96	56.1	0.04	0.96	59.6	0.03	0.96	59.5	0.03	0.96

PVE: Percent of variance explained (%) = 1-(deviance/null.deviance)

Supplementary Table 1B. Modeling the GR binding of consensus GR motifs (G-ACA---TGT-C) in different cell lines using generalized linear models (GLM)

Evaluated Parameters	3134 (1,393/8,236) +Motif PVE(%)	CV.err	AUC	3134 (1,393/8,236) −Motif PVE(%)	CV.err	AUC	AtT-20 (390/3,104) +Motif PVE (%)	CV.err	AUC	AtT-20 (390/3,104) −Motif PVE (%)	CV.err	AUC
**A1. Peak**	2.9	0.05	0.63	2.7	0.05	0.63	2.2	0.02	0.63	2.1	0.02	0.63
**A2. Background**	3	0.05	0.64	3	0.05	0.64	1.6	0.02	0.62	1.6	0.02	0.62
**A3. Relative peak**	0.2	0.05	0.53	0.2	0.05	0.53	0.4	0.02	0.56	0.4	0.02	0.56
**A. INOS (A1+A2+A3)**	**3.9**	**0.05**	**0.66**	**3.9**	**0.05**	**0.66**	**2.6**	**0.02**	**0.65**	**2.6**	**0.02**	**0.65**
**B. Overlap with CpG islands (CGIs)**	0.1	0.05	0.51	0.1	0.05	0.51	0.2	0.02	0.52	0.2	0.02	0.52
**C. Cluster of GR motifs**	0.6	0.05	0.51	-	-	-	0.1	0.02	0.50	-	-	-
**D1. Co-occurrence with canonical AP-1 motifs**	**2.6**	**0.05**	**0.57**	**2.6**	**0.05**	**0.57**	0.1	0.02	0.51	0.1	0.02	0.51
**D2. Co-occurrence with ≥2 canonical AP-1 motif**	0.3	0.05	0.51	0.3	0.05	0.51	0	0.02	0.50	0	0.02	0.50
**D3. Co-occurrence with 1-bp variants of AP-1 motif**	0.4	0.05	0.55	0.4	0.05	0.55	0	0.02	0.50	0	0.02	0.50
**D4. Co-occurrence with E-BOX motifs**	0.2	0.05	0.50	0.2	0.05	0.50	**1.1**	**0.02**	**0.55**	**1.1**	**0.02**	**0.55**
**E. Chromatin Accessibility (DHS)**	39.3	0.04	0.82	39.3	0.04	0.82	-	-	-	-	-	-
**F. c-Jun Occupancy (in c-Jun Peaks)**	18.6	0.04	0.64	18.6	0.04	0.64	-	-	-	-	-	-
**A+B**	3.9	0.05	0.66	3.9	0.05	0.66	2.6	0.02	0.65	2.6	0.02	0.65
**A+B+C**	4.2	0.05	0.67	4.2	0.05	0.67	2.7	0.02	0.65	2.7	0.02	0.65
**A+B+C+D1**	6.7	0.05	0.70	6.7	0.05	0.70	2.7	0.02	0.65	2.7	0.02	0.65
**A+B+C+D1+D2+D3+D4**	7.0	0.05	0.71	7.0	0.05	0.71	3.5	0.02	0.67	3.5	0.02	0.67
**A+B+C+D1+D2+D3+D4+E**	42.4	0.03	0.88	42.4	0.03	0.88	-	-	-	-	-	-
**A+B+C+D1+D2+D3+D4+E+F**	43.8	0.03	0.88	43.8	0.03	0.88	-	-	-	-	-	-

Supplementary Table 1C. Modeling the Hoxa2 binding to of consensus Pbx motif (TGATTGAT) using GLM

Evaluated parameters	TGATTGAT (638/59,802) PVE (%)	CV.err	AUC
**A1. Peak**	0.5	0.01	0.57
**A2. Background**	0.1	0.01	0.50
**A3. Relative peak**	0.6	0.01	0.57
**A. INOS (A1+A2+A3)**	**0.6**	**0.01**	**0.57**
**B. Overlap with CGIs**	0.0	0.01	0.51
**C. Cluster of Pbx motifs**	0.1	0.01	0.51
**D. Co-occurrence of GR-like motif (G-TGA---ATG-C)**	**0.1**	**0.01**	**0.51**
**A+B**	0.6	0.01	0.57
**A+B+C**	0.7	0.01	0.58
**A+B+C+D**	0.8	0.01	0.58

Supplementary Table 2A. Comparison of modeling the c-Jun binding to canonical AP-1 motif with 2 selected 1-bp variants using generalized linear models (GLM)

Evaluated parameters	TGCGTCA (11,926/271,973)			TGCGTCA (318/23,163)			GGACTCA (452/183,687)		
	PVE(%)	CV.err	AUC	PVE(%)	CV.err	AUC	PVE(%)	CV.err	AUC
**A1. Peak**	10.3	0.04	0.76	6.0	0.01	0.72	3.5	0.01	0.69
**A2. Background**	6.5	0.04	0.70	4.5	0.01	0.69	1.3	0.01	0.62
**A3. Relative peak**	1.9	0.04	0.62	0.6	0.01	0.58	1.0	0.01	0.61
**A. INOS (A1+A2+A3)**	**11.5**	**0.04**	**0.77**	**7.1**	**0.01**	**0.74**	**3.5**	**0.01**	**0.69**
**B. Overlap with CGIs**	0.9	0.04	0.53	6.8	0.01	0.68	1.2	0.01	0.56
**C. Cluster of AP-1 motifs**	5.5	0.04	0.66	0.1	0.01	0.50	0.1	0.01	0.50
**D. Co-occurrence of GR motifs**	**0.2**	**0.04**	**0.51**	**0.1**	**0.01**	**0.50**	**0.1**	**0.01**	**0.50**
**E. Chromatin Accessibility (DHS)**	56.5	0.04	0.93	44.7	0.01	0.95	41.4	0.01	0.95
**A+B**	12.3	0.04	0.78	9.8	0.01	0.77	4.0	0.01	0.70
**A+B+C**	13.9	0.04	0.79	10.0	0.01	0.77	4.0	0.01	0.70
**A+B+C+D**	14.0	0.04	0.79	10.0	0.01	0.77	4.0	0.01	0.70
**A+B+C+D+E**	59.6	0.04	0.96	45.1	0.01	0.95	42.7	0.01	0.95

Supplementary Table 2B. Comparison of modeling the c-Jun binding to canonical AP-1 motif (8-mer) with 3 selected control 8-mers using generalized linear models (GLM)

Evaluated parameters	ATGAGTCA (3,041/65,689)			TAATATCA (33/74,570)			TGCATCAA (47/47,424)			AGACCTGC (97/49,012)		
	PVE(%)	CV.err	AUC	PVE(%)	CV.err	AUC	PVE(%)	CV.err	AUC	PVE(%)	CV.err	AUC
**A1. Peak**	10.4	0.05	0.76	7.4	0.01	0.82	5.5	0.01	0.75	3.5	0.01	0.70
**A2. Background**	5.6	0.05	0.69	2.6	0.01	0.70	4.1	0.01	0.72	1.9	0.01	0.65
**A3. Relative peak**	2.5	0.05	0.63	2.8	0.01	0.71	1.0	0.01	0.60	0.5	0.01	0.57
**A. INOS (A1+A2+A3)**	**11.3**	**0.05**	**0.77**	**7.6**	**0.01**	**0.83**	**6.6**	**0.01**	**0.77**	**3.8**	**0.01**	**0.71**
**B. Overlap with CGIs**	0.8	0.05	0.53	0.2	0.01	0.51	0.4	0.01	0.52	0.7	0.01	0.55
**C. Cluster of AP-1 motifs**	0.8	0.05	0.53	0.0	0.01	0.50	0.1	0.01	0.51	0.0	0.01	0.50
**D. Co-occurrence of GR motifs**	**0.1**	**0.05**	**0.51**	**0.1**	**0.01**	**0.51**	**0.2**	**0.01**	**0.51**	**0.5**	**0.01**	**0.52**
**E. Chromatin Accessibility (DHS)**	55.1	0.05	0.94	40.3	0.04	0.88	44.0	0.01	0.94	42.9	0.01	0.97
**A+B**	11.4	0.05	0.77	8.1	0.01	0.84	7.5	0.01	0.78	4.0	0.01	0.72
**A+B+C**	12.2	0.05	0.78	8.1	0.01	0.84	7.6	0.01	0.79	4.0	0.01	0.72
**A+B+C+D**	12.3	0.05	0.78	8.2	0.01	0.84	7.8	0.01	0.79	4.5	0.01	0.72
**A+B+C+D+E**	56.1	0.04	0.96	43.3	0.01	0.94	47.5	0.01	0.96	44.5	0.01	0.98

Supplementary Table 2C. Comparison of modeling the GR binding to canonical GR motif with 1-bp variants using generalized linear models (GLM)

Evaluated parameters	G-ACA---TGT-C (1,393/27,176)			G-ACA---CGT-C (277/7,267)			G-ACA---TCT-C (109/61,528)		
	PVE(%)	CV.err	AUC	PVE(%)	CV.err	AUC	PVE(%)	CV.err	AUC
**A1. Peak**	2.9	0.05	0.63	1.2	0.04	0.59	1.5	0.01	0.65
**A2. Background**	3	0.05	0.64	1.2	0.04	0.59	0.4	0.01	0.59
**A3. Relative peak**	0.2	0.05	0.53	0.1	0.04	0.52	0.7	0.01	0.59
**A. INOS (A1+A2+A3)**	**3.9**	**0.05**	**0.66**	**1.7**	**0.04**	**0.60**	**1.5**	**0.01**	**0.65**
**B. Overlap with CGIs**	0.1	0.05	0.51	0.1	0.04	0.51	0.2	0.01	0.51
**C. Cluster of GR motifs**	0.6	0.05	0.51	0.1	0.04	0.51	0.1	0.01	0.50
**D. Co-occurrence of AP-1 motifs**	**2.6**	**0.05**	**0.57**	**1.7**	**0.04**	**0.57**	**2.7**	**0.01**	**0.63**
**E. Chromatin Accessibility (DHS)**	39.3	0.04	0.82	28.5	0.04	0.81	35.4	0.01	0.90
**A+B**	3.9	0.05	0.66	2.2	0.04	0.62	1.9	0.01	0.66
**A+B+C**	4.2	0.05	0.67	2.2	0.04	0.62	1.9	0.01	0.66
**A+B+C+D**	6.7	0.05	0.70	3.8	0.04	0.65	4.6	0.01	0.72
**A+B+C+D+E**	42.4	0.03	0.88	34.9	0.04	0.84	39.8	0.01	0.91

Supplementary Table 3A. Distribution of Intrinsic Nucleosome Occupancy Scores (INOS) of c-Jun motifs (TGACTCA) on the whole mouse genome

Relative Peaks	Average of PNPS in Background (-750…-150 AND +150…+750)						Total
	[-2,-1)	[-1,0)	[0,0.5)	[0.5,0.8)	[0.8,1.2)	[1.2,2)	
**[2,3)**	558(3.4%)	440(9.5%)	NA	NA	NA	NA	998(6.1%)
**[1.2,2)**	2,484(1.6%)	12,209(5.8%)	2,198(11.9%)	89(18%)	3(66.7%)	NA	16,983(6%)
**[0.8,1.2)**	1,846(0.5%)	16,923(3.7%)	10,427(9%)	**2,184(14.4%)**	378(23.3%)	5(60%)	31,763(6.2%)
**[0.5,0.8)**	1,374(0.3%)	15,197(2.6%)	13,839(6.5%)	5,766(10%)	2,596(16.9%)	125(27.2%)	38,897(6%)
**[0,0.5)**	2,001(0.1%)	23,465(1.6%)	24,128(4.6%)	13,010(7.4%)	10,464(10.7%)	1,661(14.4%)	74,729(5.1%)
**[-1,0)**	**2,204(0%)**	28,078(0.8%)	26,439(2.4%)	13,490(4.4%)	10,207(7.1%)	2,213(9.6%)	82,631(2.9%)
**[-3,-1)**	497(0%)	9,647(0.3%)	9,120(1.1%)	3,846(2.5%)	1,949(3.6%)	206(6.8%)	25,265(1.2%)
**[-5,-3)**	1(0%)	166(0.6%)	304(0.7%)	147(0.7%)	86(8.1%)	2(0%)	706(1.6%)
**Total**	10,965(0.7%)	106,125(2.3%)	86,455(4.6%)	38,532(6.7%)	25,683(9.6%)	4,212(11.9%)	**271,972(4.4%)**

Supplementary Table 3B. Distribution of Intrinsic Nucleosome Occupancy Scores (INOS) of GR-motifs (G-ACA---TGT-C) on the whole mouse genome

Relative Peaks	Average of PNPS in Background (-750…-150 AND +150…+750)						Total
	[-2,-1)	[-1,0)	[0,0.5)	[0.5,0.8)	[0.8,1.2)	[1.2,2)	
**[2,3)**	80(0%)	90(1.1%)	NA	NA	NA	NA	170(0.6%)
**[1.2,2)**	256(2%)	1,592(4.9%)	343(5.8%)	**25(12%)**	NA	NA	2,216(4.8%)
**[0.8,1.2)**	161(2.5%)	1,722(3.9%)	1,356(7.4%)	344(10.8%)	68(8.8%)	1(0%)	3,652(5.9%)
**[0.5,0.8)**	134(2.2%)	1,431(3.1%)	1,567(7.5%)	833(7.8%)	414(8.7%)	30(16.7%)	4,409(6.1%)
**[0,0.5)**	177(0.6%)	2,110(2.3%)	2,309(5%)	1,440(7.6%)	1,300(9.7%)	267(11.6%)	7,603(5.7%)
**[-1,0)**	143(0%)	2,235(1.4%)	2,356(4.2%)	1,270(5.7%)	965(8.6%)	260(8.1%)	7,229(4.3%)
**[-3,-1)**	**25(0%)**	672(1.2%)	679(3.2%)	299(4.3%)	149(10.1%)	21(14.3%)	1,845(3.3%)
**[-5,-3)**	NA	13(0%)	18(0%)	11(0%)	5(0%)	2(0%)	49(0%)
**Total**	976(1.3%)	9,865(2.8%)	8,628(5.5%)	4,222(7.1%)	2,901(9.2%)	581(10.3%)	**27,173(5.1%)**

Supplementary Table 4A. Gene Expression of Near GR ChIP-seq Peaks (<2K)

		Down-regulation (10% decrease)		No-Change		Up-regulation (10% Increase)		Total
		#	%	#	%	#	%	#
GR Peaks (1,347, 16.4%)	PRE	221	21.8%	566	55.7%	229	22.5%	1,016
	REP	60	21.1%	152	53.3%	73	25.6%	285
	UNP	11	23.9%	24	52.2%	11	23.9%	46
	Total	292	21.7%	742	55.1%	313	23.2%	1,347
GR Peaks with G-ACA---TGT-C (272, 3.3%)	PRE	14	12.0%	63	53.8%	40	34.2%	117
	REP	24	18.9%	73	57.5%	30	23.6%	127
	UNP	8	28.6%	16	57.1%	4	14.3%	28
	Total	46	16.9%	152	55.9%	74	27.2%	272
GR Peaks without G-ACA---TGT-C (1,075, 13.1%)	PRE	207	23.0%	503	56.0%	189	21.0%	899
	REP	36	22.8%	79	50.0%	43	27.2%	158
	UNP	3	16.7%	8	44.4%	7	38.9%	18
	Total	246	22.9%	590	54.9%	239	22.2%	1,075

Supplementary Table 4B. Gene Expression of Near GR ChIP-seq Peaks (<5K)

		Down-regulation (10% decrease)		No-Change		Up-regulation (10% Increase)		Total
		#	%	#	%	#	%	#
GR Peaks (2,254, 27.4%)	PRE	374	22.3%	929	55.4%	373	22.3%	1,676
	REP	120	24.4%	258	52.4%	114	23.2%	492
	UNP	14	16.3%	51	59.3%	21	24.4%	86
	Total	508	22.5%	1,238	54.9%	508	22.5%	2,254
GR Peaks with G-ACA---TGT-C (456, 5.5%)	PRE	28	14.5%	109	56.5%	56	29.0%	193
	REP	52	24.1%	122	56.5%	42	19.4%	216
	UNP	10	21.3%	28	59.6%	9	19.1%	47
	Total	90	19.7%	259	56.8%	107	23.5%	456
GR Peaks without G-ACA---TGT-C (1,798, 21.8%)	PRE	346	23.3%	820	55.3%	317	21.4%	1,483
	REP	68	24.6%	136	49.3%	72	26.1%	276
	UNP	4	10.3%	23	59.0%	12	30.8%	39
	Total	418	23.2%	979	54.4%	401	22.3%	1,798

Supplementary Table 4C. Gene Expression of Near GR ChIP-seq Peaks (<20K)

		Down-regulation (10% decrease)		No-Change		Up-regulation (10% Increase)		Total
		#	%	#	%	#	%	#
GR Peaks (4,226, 51.3%)	PRE	672	21.8%	1746	56.7%	662	21.5%	3,080
	REP	213	22.6%	522	55.3%	209	22.1%	944
	UNP	46	22.8%	119	58.9%	37	18.3%	202
	Total	931	22.0%	2,387	56.5%	908	21.5%	4,226
GR Peaks with G-ACA---TGT-C (835, 10.1%)	PRE	48	14.3%	196	58.5%	91	27.2%	335
	REP	94	23.7%	222	56.1%	80	20.2%	396
	UNP	23	22.1%	62	59.6%	19	18.3%	104
	Total	165	19.8%	480	57.5%	190	22.8%	835
GR Peaks without G-ACA---TGT-C (3,391, 41.2%)	PRE	624	22.7%	1,550	56.5%	571	20.8%	2,745
	REP	119	21.7%	300	54.7%	129	23.5%	548
	UNP	23	23.5%	57	58.2%	18	18.4%	98
	Total	766	22.6%	1907	56.2%	718	21.2%	3,391

Supplementary Table 5A. GR canonical motif (G-ACA---TGT-C) with/without co-occurrence of AP-1 canonical motif and 1-bp variants in the mouse genome and 3134 GR peaks (<150-bps)

	─ AP-1 motif				+1 AP-1 motif				≥2 AP-1 motifs			
	#	GR peak	% bound	p-value	#	GR peak	% bound	p-value	#	GR peak	% bound	p-value
**0 variants**	10,201	364	3.6%	-	590	90	15.3%	NA	-	-	-	-
**1 variant**	9,329	437	4.7%	9.9E-05	544	79	14.5%	0.79	-	-	-	-
**2 variants**	4,274	217	5.1%	3.0E-05	369	71	19.2%	0.13	-	-	-	-
**3 variants**	1,343	82	6.1%	8.2E-06	-	-	-	-	-	-	-	-
**≥4 variants**	473	38	8.0%	1.2E-06	-	-	-	-	-	-	-	-
**Total**	25,620	1,138	4.4%	-	1,503	240	16.0%	< 2.2E-16	53	15	28.3%	8.52E-16

Supplementary Table 5B. 1-bp variant of GR canonical motif (G-ACA---AGT-C) with/without co-occurrence of AP-1 canonical motif and 1-bp variants in the mouse genome and 3134 GR peaks (<150-bps)

	─ AP-1 motif				+1 AP-1 motif				≥2 AP-1 motifs			
	#	GR peak	% bound	p-value	#	GR peak	% bound	p-value	#	GR peak	% bound	p-value
**0 variants**	16,789	169	1.0%	-	953	47	4.9%	-	-	-	-	-
**1 variant**	15,660	225	1.4%	4.9E-04	924	61	6.6%	0.15	-	-	-	-
**2 variants**	7,492	130	1.7%	2.7E-06	652	47	7.2%	0.07	-	-	-	-
**3 variants**	2,367	50	2.1%	3.6E-06	-	-	-	-	-	-	-	-
**≥4 variants**	720	14	1.9%	2.5E-02	-	-	-	-	-	-	-	-
**Total**	43,028	588	1.4%	-	2,529	155	6.1%	< 2.2E-16	84	16	19.0%	< 2.2E-16

Supplementary Table 5C. GR canonical motif (G-ACA---TGT-C) with/without co-occurrence of AP-1 and E-Box canonical motif in the mouse genome and AtT-20 GR peaks (<150-bps)

	Genome	GR peak	%bound	p-value
**─ AP-1 motif**	25,620	359	1.4%	-
**+1 AP-1 motif**	1,503	31	2.1%	0.05
**≥2 AP-1 motifs**	53	0	0.0%	0.78
**─ E-Box motif**	25,373	327	1.3%	-
**+1 E-Box motif**	1,444	45	3.1%	1.5E-08
**≥2 E-Box motifs**	359	18	5.0%	4.6E-09
**Total**	27,176	1,393	5.1%	-
